# Selective RNA Processing and Stabilization are Multi‐Layer and Stoichiometric Regulators of Gene Expression in *Escherichia coli*

**DOI:** 10.1002/advs.202301459

**Published:** 2023-10-16

**Authors:** Daixi Liu, Haibo Lv, Yafei Wang, Jinyu Chen, Dexin Li, Ranran Huang

**Affiliations:** ^1^ Institute of Marine Science and Technology Shandong University 72 Binhai Road Qingdao Shandong 266237 China; ^2^ School of Pharmaceutical Sciences Shandong University 44 Wenhuaxi Road Jinan Shandong 250012 China; ^3^ School of Computer Science and Technology Shandong University 72 Binhai Road Qingdao Shandong 266237 China

**Keywords:** selective RNA processing and stabilization, simultaneous 5′ and 3′ end sequencing, stem‐loop structure, stoichiometric regulation, targetome

## Abstract

Selective RNA processing and stabilization (SRPS) facilitates the differential expression of multiple genes in polycistronic operons. However, how the coordinated actions of SRPS‐related enzymes affect stoichiometric regulation remains unclear. In the present study, the first genome‐wide targetome analysis is reported of these enzymes in *Escherichia coli*, at a single‐nucleotide resolution. A strictly linear relationship is observed between the RNA pyrophosphohydrolase processing ratio and scores assigned to the first three nucleotides of the primary transcript. Stem‐loops associated with PNPase targetomes exhibit a folding free energy that is negatively correlated with the termination ratio of PNPase at the 3′ end. More than one‐tenth of the RNase E processing sites in the 5′‐untranslated regions（UTR） form different stem‐loops that affect ribosome‐binding and translation efficiency. The effectiveness of the SRPS elements is validated using a dual‐fluorescence reporter system. The findings highlight a multi‐layer and quantitative regulatory method for optimizing the stoichiometric expression of genes in bacteria and promoting the application of SRPS in synthetic biology.

## Graphical abstract

The selective RNA processing and stabilization (SRPS) facilitate differential expression of multiple genes in polycistronic operons. The discovery presents a multilayer, quantitative, and ubiquitous posttranscriptional regulatory mechanism as well as elements to precisely optimize gene expression stoichiometry. Moreover, this finding suggests a novel strategy of ‘polycistronic operon plus SRPS elements’ to address the challenge of ‘balanced expression of multiple genes in chassis cells’ in synthetic biology.

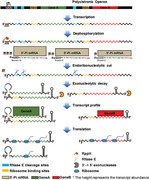

## 1 Introduction

Natural products are compounds with great value in the biological, pharmaceutical, and industrial fields. However, many difficulties are encountered when culturing the original strains of natural products. Synthetic biology, which involves cloning synthetic gene clusters into chassis cells, is an important method for synthesizing natural products. Synthetic gene clusters are often cloned into host cells such as *Escherichia coli* for heterologous expression. However, there is also a need for the introduction of multiple genes encoding metabolic pathway enzymes into chassis microorganisms for the synthesis of natural and synthetic products.^[^ ^1^, ^2^, ^3^ ^]^ To prevent the accumulation of hazardous intermediates or bottlenecks that result in growth inhibition or inadequate yield, genes must be expressed at adequately balanced levels to produce these compounds at commercially relevant levels. Similarly, because excess subunits that do not have binding partners may misfold or aggregate and drive proteotoxicity,^[^ ^4^ ^]^ manipulation of multi‐subunit proteins (such as cellulosomes,^[^ ^5^ ^]^ ion channels,^[^ ^6^ ^]^ and photosystem complexes)^[^ ^7^ ^]^ frequently necessitates the coordinated expression of several genes to produce subunits at the appropriate stoichiometries.^[^ ^8^ ^]^ A key challenge in synthetic biology is the development of precisely regulated and well‐characterized molecular components to construct optimal biological pathways or functioning protein complexes. It is nearly impossible to predict the strengths of promoters and ribosome‐binding sites (RBSs) required to balance and coordinate the expression of multiple genes.^[^ ^9^ ^]^

Organizing multiple related genes into operons, as prokaryotes naturally do,^[^ ^10^ ^]^ is a practical strategy to simultaneously regulate several genes.^[^ ^11^ ^]^ Moreover, it reduces noise in stochastic gene expression,^[^ ^12^ ^]^ facilitates complex assembly,^[^ ^13^ ^]^ and minimizes the demand for gene transcriptional resources.^[^ ^14^ ^]^ The relative expression of each open reading frame (ORF) in the operon is then set by altering post‐transcriptional processes such as selective RNA processing and stabilization (SRPS),^[^ ^15^ ^]^ where the primary mRNA transcribed as an operon is first processed by nucleases into segments, and stability variations among the segments contribute to differential gene expression. Unlike transcriptional termination, which generates only a staircase‐like transcript abundance (TA) pattern, SRPS leads to more complex TA patterns. For example, it has been reported that 12 genes within the *Clostridium cellulolyticum cip‐cel* operon, which is regulated by SRPS, exhibit highly skewed TAs (ratios of 100:110.2:8.6:8.1:38.1:5.0:3.9:2.0:2.6:2.2:3.2:4.7).^[^ ^16^ ^]^ SRPS has been reported in several operons of gram‐negative^[^ ^17^, ^18^, ^19^, ^20^, ^21^, ^22^, ^23^, ^24^, ^25^ ^]^ and gram‐positive^[^ ^26^, ^27^ ^]^ bacteria. The differential RNA sequencing (RNA‐seq)^[^ ^28^ ^]^ technique, which has been used to unveil the first global landscape of SRPS regulation in *C. cellulolyticum*,^[^ ^16^ ^]^ suggests that SRPS is a conserved global regulatory mechanism. However, the quantitative regulation of TA by SRPS remains unknown, which seriously hampers its application in synthetic biology.

Ribonucleases (RNases) are key players in SRPS,^[^ ^29^ ^]^ and are divided into two main classes: endoribonucleases (endoRNases) and exoribonucleases (exoRNases), which regulate RNA stability by cleaving the RNA internally or attacking RNA from either its 5′ or 3′ end, respectively. Although the transcription and translation processes may be quite similar in different bacteria, their RNase activities are different. EndoRNases initiate the SRPS. RNase E, an important endoRNase in *E. coli*,^[^ ^30^ ^]^ with orthologs widely distributed in many species, including bacteria, cyanobacteria, and plant chloroplasts.^[^ ^31^ ^]^ However, there is no ortholog of RNase E in *Bacillus subtilis* RNases Y and J play critical roles in regulating the abundance of mRNAs in *B. subtilis*.^[^ ^32^ ^]^ Besides, RNase III is a unique endoRNase that shears RNA within double‐stranded regions.^[^ ^33^ ^]^ Furthermore, exoRNases play a complementary role by rapidly degrading decay intermediates. ExoRNases are divided into two main categories: 3′‐to‐5′ and 5′‐to‐3′ exonucleases. There are several 3′‐to‐5′ endoRNases (PNPase, RNase II, and RNase R) in *E. coli*. However, there is no 5′‐to‐3′ endoRNase in *E. coli*. The first 5′‐to‐3′ endoRNase, RNase J, was identified in *B. subtilis*.^[^ ^34^ ^]^ The RNase J family of proteins is widely distributed in archaeal and bacterial species. For example, RNase J in *Synechocystis* PCC 6803 participates in the response to environmental stress.^[^ ^35^ ^]^

*E. coli* is an important model microorganism that is widely used as a host for the biosynthesis of specialty chemicals in pharmaceutical, chemical, and agricultural applications. The model of the SRPS process in *E. coli* is as follows: The primary endoRNase that initiates the SRPS process in *E. coli* is RNase E (encoded by *rne*), whose endonucleolytic activity at the internal sites is greatly enhanced as the 5′‐triphosphate of the transcript is converted to 5′‐monophosphate by RNA pyrophosphohydrolase (RppH; encoded by *rppH*).^[^ ^36^, ^37^, ^38^ ^]^ Several 3′‐to‐5′ exoribonucleases (PNPase, RNase II, and RNase R, encoded by *pnp*, *rnb*, and *rnr*, respectively) then digest the products originating from the initial cleavage.^[^ ^39^, ^40^, ^41^ ^]^ Among these, the stem‐loop structure plays a key role in RNA stability; it is generally located in the 3′‐untranslated region and 5′‐UTR of mRNA, and its stability affects the processing efficiency of nucleic acid exonucleases. The stability of the stem‐loop is determined by ΔG.^[^ ^42^, ^43^ ^]^ With the emergence of RNA‐seq techniques that allow for the global detection of RNase cleavage sites, several targetomes of endoRNases and exoRNases have been determined, such as RNase E in *Salmonella enterica*;^[^ ^44^ ^]^ RNase Y in *Streptococcus pyogenes*,^[^ ^45^ ^]^ *Staphylococcus aureus*,^[^ ^46^ ^]^ and *B. subtilis*;^[^ ^27^ ^]^ and 3′‐to‐5′ exoRNases in *S. pyogenes*.^[^ ^47^ ^]^ Nevertheless, the genome‐wide targets of RppH, endoRNases, and exoRNases in *E. coli* and the mechanisms by which these enzymes interact to quantitatively regulate gene expression have never been investigated.

Using *E. coli* str. K‐12 substr. MG1655 as a model and its SRPS‐related gene mutants (the Δ*rne*‐3071(ts), Δ*pnp*, Δ*rnb*, and Δ*rnr* strains; we used published transcriptome data for these),^[^ ^48^ ^]^ we first revealed the genome‐wide targetomes of these enzymes in *E. coli* at a single‐nucleotide resolution, by integrating the methods of simultaneous 5′ and 3′ end sequencing (SEnd‐seq),^[^ ^48^ ^]^ identification of specific cleavage positions [ISCP; wild‐type (WT) vs RNase‐deficient strain].^[^ ^49^ ^]^ Following that, we revealed the interplay between RppH, endoRNase (RNase E), and exoRNases (PNPase, RNase II, and RNase R). We quantitatively characterized SRPS processes and depicted a quantitative regulation model of SRPS in *E. coli*. At the post‐transcriptional layer, the process efficiency (pyrophosphate removal ratio) of RppH is regulated by the first three nucleotides at the 5′ end of the primary transcript, and thus determines the cleavage efficiency of RNase E by the cascade; the stability of the stem‐loops formed by the 3′ nucleotide sequences of the processed mRNA fragments positively affects the termination of the 3′‐to‐5′ exoRNase activities. These synergistic actions collectively modulate the TA in *E. coli*. At the translational layer, different endoRNase cleavage sites in the 5′‐UTR lead to differential stem‐loop formation in the 5′‐UTR, which affects ribosome‐binding and thus modulates translational initiation efficiency. The effectiveness of these SRPS elements was validated using a dual‐fluorescence reporter system. To further explore and utilize SRPS elements, we constructed the first database of prokaryotic SRPS sites (ProSRPSite, http://prosrpsite.sdu.edu.cn/). Thus, our findings revealed the molecular mechanism of SRPS and identified a multi‐layered, quantitative, and ubiquitous regulatory mechanism. The quantification of these SRPS elements allows for their application in precisely optimizing the stoichiometric expression of target genes in synthetic biology.

## 2 Results

### 2.1 Targetomes of RppH, endoRNase (RNase E), and exoRNases (PNPase, RNase II, and RNase R) in *E. coli*

**Figure 1:**
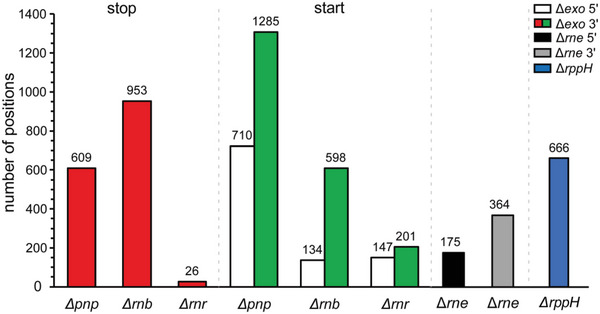
The number of RppH, RNase E, and exoRNase (PNPase, RNase II, and RNase R) processing positions identified. Schematic representation of the numbers of trimming stop (red) and start (green) positions for each exoRNase. The numbers of positions processed by RNase E and RppH are shown in the gray/black and blue columns, respectively.

To investigate the action of SRPS‐related enzymes on transcripts in *E. coli*, we first detected their genome‐wide targetomes (SRPS enzymes) (Figure S1, Supporting Information). The processed WT library was extracted from the total library using a special 5′‐adapter sequence which was ligated to the 5′‐P_i_ RNA. Following that, with the help of the method used for identifying the transcriptional start sites (TSSs) in the SEnd‐seq data,^[^ ^48^ ^]^ 666 positions processed by RppH were identified (**Figure** **1**; Figure S1a and Table S1, Supporting Information). Using an integration of the SEnd‐seq and ISCP methods,^[^ ^48^, ^49^ ^]^ 539 positions processed by RNase E (175 5′ and 364 3′ *rne* ends) were identified. In addition, positions 2604, 1685, and 374 were trimmed using PNPase, RNase II, and RNase R, respectively (Figure 1; Figure S1b,c, Tables S2 and S3, Supporting Information).

There were conserved –10 elements (TATAAT) upstream of the RppH targetome (Figure S2a, Supporting Information). Although there was no consensus sequence downstream in general, the first RNA nucleotide preferred to be a consensus AMP as the processing ratio increased (Figure S2a, Supporting Information; Experimental Section, details are discussed in Section 4). These results are consistent with those of a previous in vitro study showing that RppH has a modest preference for A at the 5′ end, when triggering the conversion of 5′‐PPP_i_ RNA to 5′‐P_i_.^[^ ^50^ ^]^

**Figure 2:**
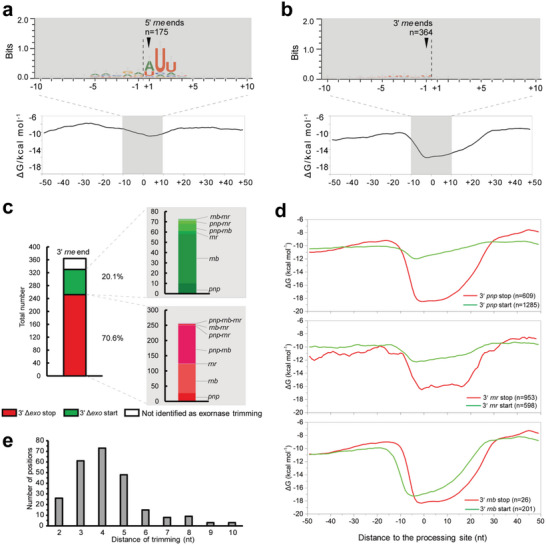
Analysis of the sequences and structure conservations of the identified targetomes. a,b) Sequence and structure conservation of the 5′ (a) and 3′ (b) *rne* ends. The figure was created after alignment of all sequences from 10 nt on each side of the identified ends. Error bars indicate 95% confidence intervals. The average of the minimal free energy (Δ*G*/kcal·mol^−1^) was calculated at each position using a sliding window of 50 nt located upstream of the identified ends. c) The portions of the 3′ *rne* ends corresponding to the 3′ Δ*exo* stop (bottom portion), 3′ Δ*exo* start (middle portion), and not associated with 3′‐to‐5′ exoRNases trimming positions (top portion). d) Structure conservation at the 3′ trimming start and stop positions of PNPase, RNase II, and RNase R. Δ*G* was calculated using a sliding window of 50 nt located upstream of each position. e) Distribution of the trimming distances between 3′ *rnb* start and stop positions located within 10 nt.

Distinct features were observed between the 5′ and 3′ *rne* ends of the RNase E targetome. First, there was a conserved motif “RN↓WUU” (where R is G/A, W is A/U, N is any nucleotide, and ↓ is the site of cleavage) around the 5′ *rne* end, which was in accordance with previous findings (**Figure** **2**a).^[^ ^44^ ^]^ The situation at the 3′ *rne* end was quite different, as no conservative motifs were observed (Figure 2b). Moreover, the preference of RNase E for nucleotides was distinct from that of RNase Y, which preferentially cleaves after a G.^[^ ^45^ ^]^ Second, there was a significant decrease in the minimum free energy (MFE) upstream of the 3′ *rne* end, indicating the existence of a putative RNA structure (Figure 2b). In contrast, the MFE in the proximity of the 5′ *rne* end was stable and remained at a high level, indicating that these sequences corresponded to single‐stranded RNA regions (Figure 2a). Additionally, some 5′ *rne* ends were confirmed by means of 5′ rapid amplification of cDNA ends (RACE) PCR (Figure S3, Supporting Information).

Moreover, the 3′ *rne* end was compared with the trimming positions of three 3′‐to‐5′ exoRNases: PNPase, RNase II, and RNase R (Experimental Section). A total of 20.1% of 3′ *rne* ends corresponded to 3′‐to‐5′ exoRNase trimming start positions, while 70.6% of 3′ *rne* ends corresponded to stop positions (Figure 2c). This indicates that most of the transcripts sheared by RNase E were subsequently trimmed by 3′‐to‐5′ exoRNases whose action stopped at a stable stem‐loop, thus leading to a low MFE and the absence of a conserved motif near the 3′ *rne* end. Furthermore, no conserved motifs were observed near the trimming start and stop positions of the three 3′‐to‐5′ exoRNases (Figure S2b–d, Supporting Information).

Structural analysis of the three exoRNase targetomes showed that there was a decrease in the average MFEs around the 3′ *exornases* start and stop sites. Interestingly, there was only a modest decrease in the MFE around the 3′ *pnp* start, whereas that around the 3′ *pnp* stop site decreased significantly, by nearly 90% (Figure 2d). This indicates that the stem‐loops formed upstream of the 3′ *pnp* start site are more vulnerable to attack by PNPase than those formed around the 3′ *pnp* stop, because the activity of PNPase in this case is blocked by a stable stem‐loop.^[^ ^51^ ^]^ The situation for RNase R was similar to that for PNPase. In case of RNase II, however, the situation was somewhat different, and the MFE around the 3′ *rnb* start and stop sites decreased significantly. In addition, there was a small range of offsets (3–5 nt) between the minimum MFE values of the 3′ *rnb* start and stop sites (Figure 2d).

### 2.2 The Functions and Mechanisms of RNases in TA Regulation

A total of 598 and 953 trimming start and stop positions of RNase II, respectively, were identified (Table S3, Supporting Information). Two hundred and fifty‐four 3′ *rnb* start sites were located less than 10 nt downstream of the trimming stop positions, with 182 of these 254 at a distance of 3–5 nt, which was consistent with the offset between the MFEs around the 3′ *rnb* start and stop sites (Figure 2d,e; Table S4, Supporting Information). This indicates that RNase II has a low processivity, similar to that of YhaM in *S. pyogenes*.^[^ ^47^ ^]^

**Figure 3:**
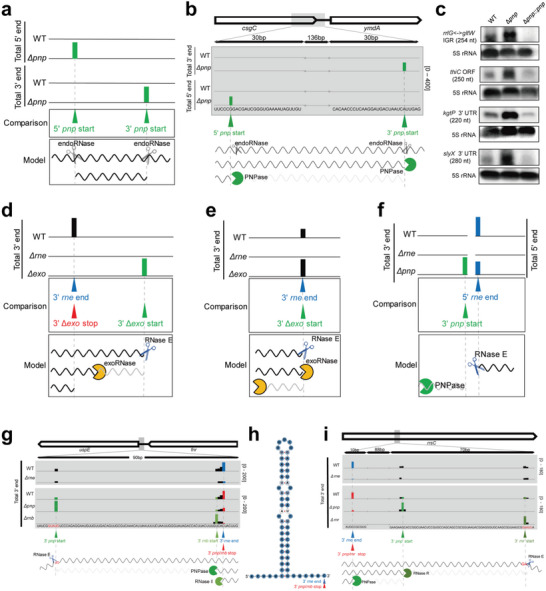
Mechanisms underlying the interplay between endoRNases and exoRNases in transcript decay. a) Mechanism and b) schematic of the fragment generated by endoRNase and further fully degraded by PNPase in the WT. Briefly, endoRNase sheared the transcript and generated a short fragment, following which the PNPase digested the fragment from its 3′ end until it was fully degraded, with the 5′ end of the fragment identified as the 5′ *pnp* start. c) There were four fragments, as confirmed using northern blot. d–f) Model for the in vivo endoRNase and exoRNase degradation of the transcripts. The upper panel shows an example of transcript 5′ and 3′ end coverage profiling using RNA sequencing. The middle panel shows the RNA ends processed by RNases. The RNA ends corresponding to RNase E cleavage positions are indicated using blue arrowheads. The RNA ends corresponding to the trimming start and stop positions of exoRNases are indicated using green and red arrowheads, respectively. The bottom shows the model in which the RNA was processed by RNases. g,h) RNase E sheared the transcript at the IGR between *uspE* and *fnr*, and the exposed 3′ end was further digested by PNPase until a stable stem‐loop (k) was encountered. RNase II further nibbled the tail of the stem‐loop. i) RNase E cleaved at the ORF of *rrsC*. Fifty‐eight nt at the exposed 3′ end were digested by RNase R, while 99 nt were further degraded by PNPase. The nucleotides highlighted in red correspond to the consensus sequence of the RNase E processing site.

Furthermore, 1285 3′ trimming start positions of PNPase were identified, which were more than twice the number of stop positions (*n* = 609), indicating that most targets of PNPase were fully degraded. Here, the 5′ end of the short fragments, which are generated by an endoRNase and fully degraded from its 3′ end by PNPase, was identified as the 5′ *pnp* start (**Figure** **3**a). The 5′ *rnb* start and 5′ *rnr* start sites were defined similarly. Indeed, we detected a large number of 5′ *pnp* start sites (*n* = 710), as compared to 5′ *rnb* (*n* = 134) or 5′ *rnr* (*n* = 147) start sites. In total, 125 5′ *pnp* start sites were located 50–300 nt upstream of the 3′ *pnp* start sites (Table S5, Supporting Information), indicating that short fragments sheared by endoRNases are the major substrates of PNPase. Fragments generated by endoRNases and fully degraded by PNPase were identified (Figure 3a,b; Figure S4 and Table S5, Supporting Information). For instance, fragments originating from the intergenic region (IGR) of *rrlG* and *gltW*, the ORF of *thiC*, and the 3′‐UTR of *kgtP* and *slyX* were confirmed using northern blotting (Figure 3c). The real read coverage for the fragments was visualized using Integrative Genome Viewer (Figure S5, Supporting Information).

In contrast, RNase R showed limited activity in *E. coli*. Although 201 and 26 trimming start and stop positions, respectively, were identified for RNase R, there were only four processing events in which both trimming start and stop positions were detected (Table S4, Supporting Information). Moreover, only 11 fragments accumulated in the absence of RNase R (Table S5, Supporting Information).

To investigate how RNase E cooperates with the 3′‐to‐5′ exoRNases to regulate TA, we compared the targetomes of RNase E and three exoRNases. The mechanisms were as follows: a) The transcript was first sheared by RNase E, following which the 3′‐to‐5′ exoRNases digested the transcript from the exposed 3′ end and stopped at the 3′ *rne* end (Figure 3d; Table S6, Supporting Information); b) The decay intermediates were first sheared by RNase E, following which the exoRNases digested them from the 3′ *rne* end (Figure 3e; Table S6, Supporting Information); c) The transcript was first sheared by RNase E, following which PNPase trimmed the transcript upstream of the 5′ *rne* end (Figure 3f; Table S6, Supporting Information). Furthermore, various exoRNases acted synergistically after the cleavage of RNase E. In one case, PNPase digested RNA, starting at 81 nt from the 3′ end sheared by RNase E, and RNase II then bled 4 nt at the end of the stem‐loop (Figure 3g,h). In the other case, RNase R digested an RNA, starting at 58 nt from the 3′ end of the transcripts obtained after RNase E cleavage, following which PNPase digested the remaining 99 nt (Figure 3i).

### 2.3 The Targetomes of SRPS Enzymes are Mainly Located in the Complex‐Operon

According to the updated definitions of operons and transcription units,^[^ ^52^ ^]^ we concluded that 588 polycistronic operons in *E. coli* utilized transcript unit information.^[^ ^48^ ^]^ To investigate the effects of SRPS enzymes on polycistronic transcripts, their processing positions inside each polycistronic operon were determined (Table S7, Supporting Information). As expected, more than 50% were located within polycistronic operons, especially the 5′ *rne* end and 3′ *rnr* stop site (over 80%) (**Table** **1**). Furthermore, based on the Fragments Per Kilobase per Million mapped fragments (FPKM) ratios of adjacent genes, polycistronic operons exhibiting a highly skewed TA landscape (complex‐operons) were identified (Experimental Section). More than 70% of the processing positions within the polycistronic operons were located in the complex‐operon, which accounted for only 50% of all polycistronic operons (Table 1). These findings suggested that these enzymes primarily use SRPS to control TA in a complex‐operon, to achieve optimal gene expression.

**Table 1:** Distribution of SPRS‐enzyme targetomes on operons.

Number	Total	Polycistronic operon	Complex‐operon
Type of Position			
*RppH*	666	418 (63%)	288 (69%)
5′ *rne* end	175	145 (83%)	120 (83%)
3′ *rne* end	364	204 (56%)	163 (80%)
3′ *pnp* stop	609	338 (56%)	238 (70%)
3′ *pnp* start	1285	711 (55%)	495 (70%)
5′ *pnp* start	701	343 (49%)	240 (70%)
3′ *rnb* stop	953	557 (58%)	385 (69%)
3′ *rnb* start	598	275 (46%)	212 (77%)
5′ *rnb* start	134	82 (61%)	69 (84%)
3′ *rnr* stop	26	21 (81%)	16 (76%)
3′ *rnr* start	201	102 (51%)	78 (76%)
5′ *rnr* start	147	55 (37%)	40 (73%)

The percentages in the table have been calculated as the current column divided by the previous column on the same line.

### 2.4 The Nucleotide Sequences and Structures Modulate the Stability and Abundance of the Transcript

**Figure 4:**
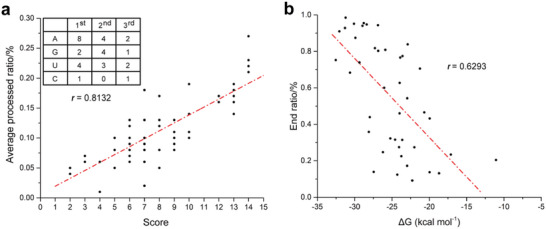
The actions of RppH and PNPase on the quantitative modulation of the TA. a) The table in the top‐left corner presents the scores assigned to the first three RNA nucleotides of the TSS. The scatter diagram shows the correlation between the average processing ratio and sum of the scores assigned to the three nucleotides. b) The scatter diagram shows the correlation between the end ratios at the trimming stop positions of PNPase and the MFE (Δ*G*/kcal·mol^−1^), which were predicted from a window 50 nt upstream of the same positions. TA, transcript abundance; TSS, transcriptional start site.

While RppH requires at least two unpaired nucleotides, it prefers three or more nucleotides, as determined by several *in vitro studies* ^[^ ^50^, ^53^, ^54^ ^]^ and has a sequence preference for the 5′ ends of its substrates. To quantitatively investigate the function of RppH in SRPS, we evaluated the relationship between the first three nucleotides of TSS and the ratio of transcript processing by RppH. We assigned an appropriate score to the first three RNA nucleotides at RppH processing sites based on sequence preferences in vitro^[^ ^50^, ^53^, ^54^ ^]^ and observed a noticeable positive correlation (**Figure** **4**a, *r* = 0.8132, Experimental Section; Table S1, Supporting Information). This indicates that the first three RNA nucleotides at the 5′ end of the transcript determine the ratio of transcript processed by RppH. The cleavage efficiency of RNase E was affected because its affinity for 5′‐P_i_ RNA was significantly higher (over 20‐fold) than that for 5′‐PPP_i_ RNA.^[^ ^38^ ^]^

The Δ*G* of the stem‐loop as determined by the 3′ end sequence varied widely, which quantitatively determined the associated TA ratio in *C. cellulolyticum*.^[^ ^16^ ^]^ In addition, the stem‐loop structure protects transcripts from degradation by 3′‐to‐5′ exoRNases such as PNPase and RNase II in *E. coli*.^[^ ^55^ ^]^ In the present study, we investigated whether this quantitative relationship exists in *E. coli* and the underlying practical molecular mechanism. First, we selected 42 3′ *pnp* stops in which there was a 3′ *pnp* start located less than 300 nt downstream. Second, the correlation between the end ratio at the 3′ *pnp* stop and the Δ*G* of stem‐loops upstream of the same position was assessed, which was negative (Figure 4b, *r* = 0.6293, Experimental Section; Table S4, Supporting Information). In summary, these results indicated that the more stable the stem‐loop formed on the 3′ end of the transcript, the less susceptible it is to degradation by PNPase, thus resulting in a higher TA.

### 2.5 RNase E Regulates Translation Efficiency via 5′‐UTR Processing

More than one‐third (66/175) of the 5′ *rne* ends were located in operons that were cleaved by RNase E at least twice in their 5′‐UTR or in the same IGR (Experimental Section; Table S8, Supporting Information). As a result, the transcript downstream of the 5′ *rne* end had 5′‐UTRs with distinct lengths. Among the 43 operons described above, 33 contained more than one 5′ *rne* ends located in their 5′‐UTR, while 10 were cleaved by RNase E at least twice in the same IGR. Interestingly, secondary structure prediction revealed that the covered RBS on the 5′‐UTR of 13 transcripts (containing twenty‐one 5′ *rne* ends) was exposed when RNase E was cleaved at the position closest to the 5′ boundary of the downstream ORF (Figure S6, Supporting Information). These results suggest that the different 5′‐UTRs on transcripts form different secondary structures, resulting in an exposed or covered RBS, thus affecting the affinity of the RBS for the ribosome and further regulating the translational initiation rate.

**Figure 5:**
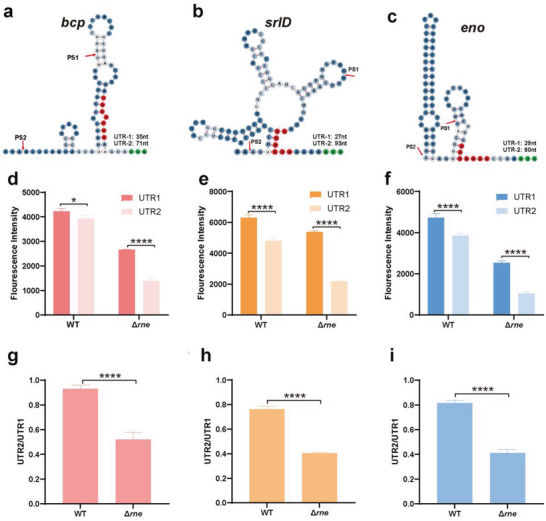
5′‐UTR processing exposed the RBS on the transcript and enhanced the translation efficiency. Stem‐loops formed by the 5′‐UTR‐2 sequences of a) *bcp*, b) *srlD*, and c) *eno*. The red arrow indicates the two processing sites (PS1 and PS2) of RNase E. The red and green nucleotides represent the RBS and translation initiation codon, respectively. d–f) A schematic depicting the fluorescence intensities measured by the mCherry reporter system constructed by the insertion of the 5′‐UTR‐1 and 5′‐UTR‐2 sequences of *bcp* (d), *srlD* (e), and *eno* (f). g–i) The normalized mCherry fluorescence intensity with inserted 5′‐UTR‐2 of *bcp* (g), *srlD* (h), and *eno* (i). Two‐tailed Student's t‐test is applied to detect differences between two groups. *p < 0.05, ****p < 0.0001. Data represent the mean ± s.d. of at least three biological replicates.

Three examples (*eno*, *bcp*, and *srlD*) were selected to test this hypothesis (**Figure** **5**a–c). The mCherry reporter system was constructed and transformed into Δ*rne*‐3071(ts) (Figure S7, Supporting Information). The *mcherry* gene was ligated to these different 5′‐UTRs and mCherry fluorescence intensities were monitored to assess translation efficiency. Our data showed that the shorter 5′‐UTR (UTR‐1; where the RBS was exposed) ligated to *mcherry* resulted in higher fluorescence intensity. All three cases showed that the mCherry fluorescence intensity with a shorter 5′‐UTR was higher than with a longer 5′‐UTR (UTR‐2) both in the WT and the Δ*rne*‐3071(ts) (Figure 5d–f, Supporting Information). Furthermore, in order to test how much RNase E cleavage changed the expression of these three genes, the UTR2‐data from each strain was normalized with the signal from their respective UTR‐1(Figure 5g–i, Supporting Information). The results indicated that the normalized mCherry fluorescence intensity with inserted 5′‐UTR‐2 in WT was relatively higher compared to the Δ*rne*‐3071(ts) strain. The probable reason would be the partial shearing of UTR‐2 by the RNase E in WT, which resulted in a short UTR and therefore induced a higher expression. In summary, RNase E cleavage at different positions on the 5′‐UTRs regulates their translation efficiency.

### 2.6 The SRPS Stoichiometric Regulatory Model in *E. coli*

Based on the above conclusions, we propose the following quantitative regulatory model for SRPS in *E. coli*: After the genes within the operon are transcribed simultaneously, RppH catalyzes the removal of pyrophosphate from the 5′‐terminus of the primary transcripts, and the processing efficiency (pyrophosphate removal ratio) of RppH is determined by the first three RNA nucleotides at the 5′‐terminus. After the initial endonucleolytic cleavage by RNase E, which generates SRPS intermediates with 5′‐monophosphate ends, RNase E can proceed in the 5′‐to‐3′ direction, generating additional processed transcripts. Because the affinity of RNase E for 5′‐monophosphate RNA is significantly higher (over 20‐fold) than that for 5′‐triphosphate RNA,^[^ ^38^ ^]^ the first three RNA nucleotides on the 5′‐terminus of the primary transcript determine the RNase E cleavage efficiency by the cascade. The RNase E‐processed transcripts are then degraded by exoRNases (PNPase, RNase II, and, in some cases, RNase R), and the stability of the stem‐loop formed by 3′ nucleotide sequences on RNase E‐processed transcripts determines the termination efficiency of exoRNases at the 3′ terminus. This synergistic action ultimately modulates the TA in *E. coli*. At the translational layer, cleavage of the 5′‐UTR by RNase E leads to the formation of different stem‐loops, which affects the affinity of the RBSs for the ribosome and thus modulates the translation efficiency.

### 2.7 Validation of SRPS Applications in Synthetic Biology

**Figure 6:**
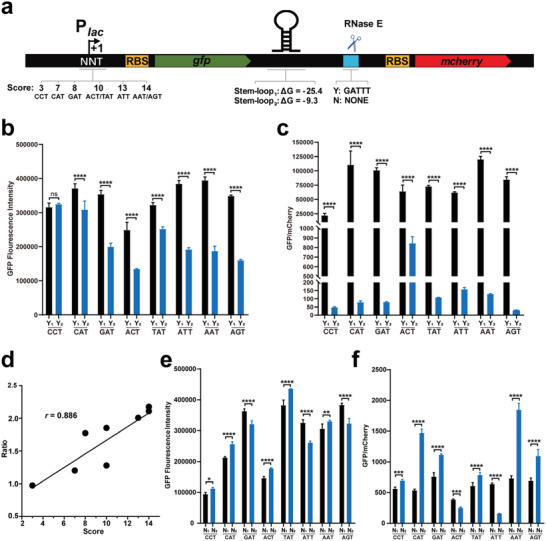
The feasibility of using SRPS elements in synthetic biology applications was confirmed in vitro. a) Schematic representation of the dual‐fluorescence reporter system consisting of a *lac* promoter; two reporter genes, *gfp* and *mcherry*; eight TSSs with different scores; two stem‐loops (Δ*G* = −25.4 and −9.3 kcal mol^−1^); and an RNase E cleavage site (GATTT). b,c) When the RNase E cleavage site was added, there was variation in the GFP fluorescence intensity (b) and GFP/mCherry ratio (c) upon changing the stem‐loops (Y_1_ vs Y_2_) and the first two RNA nucleotides of the TSS. d) Correlation between the sum of TSS scores and ratio of *gfp* expression upon addition of the two different stem‐loops. e,f) The impact of removal of RNase E cleavage sites and change of stem‐loops (N_1_ vs N_2_) on the GFP fluorescence intensity (e) and GFP/mCherry ratio (f). Y_1_/Y_2_ represents stem‐loop_1_/stem‐loop_2_ removed and RNase E cleavage site inserted. N_1_/N_2_ represents stem‐loop_1_/stem‐loop_2_ inserted and RNase E cleavage site removed. Two‐tailed Student's t‐test is applied to detect differences between two groups. ****p < 0.0001, ***p < 0.001, **p < 0.01, and ns represents not significant. Data has been represented as mean ± s.d. of at least three biological replicates.

To assess the feasibility of using SRPS elements in synthetic biology, we constructed a dual‐reporter system (**Figure** **6**a). Considering that the action of RppH was most influenced by the first two nucleotides at the 5′‐terminus, CCT, CAT, GAT, ACT, TAT, ATT, AAT, and AGT were determined to be the first three nucleotides of the TSS (scores from 3 to 14), to assess the effect of RppH on the TA. Stem‐loops with Δ*G* values of −25.4 (stem‐loop_1_) and −9.3 (stem‐loop_2_) kcal·mol^–1^ were individually inserted between the reporter genes *gfp* and *mcherry*, to assess their effects on exoRNase processivity. To assess the influence of RNase E cleavage on the TA, the RNase E cleavage site was located between the stem‐loops and *mcherry*. When the RNase E cleavage site was inserted, the more stable stem‐loop (Δ*G* = −25.4 vs −9.3 kcal·mol^–1^) significantly enhanced the expression of the *gfp* that was located upstream of the stem‐loop (Figure 6b). Moreover, as the TSS score increased, the difference in *gfp* expression between the stem‐loop_1_ (Y_1_) and stem‐loop_2_ (Y_2_) expanded (Figure 6b). There was an apparent positive correlation between the TSS scores and ratio of GFP fluorescence intensity between stem‐loop_1_ and stem‐loop_2_ (Y_1_ vs Y_2_) (Figure 6d, *r* = 0.886). Because a higher TSS score corresponds to a higher ratio of transcripts processed by RppH, the difference in *gfp* expression caused by the stability of the stem‐loop is related to the processing efficiency of RppH. This could be attributed to the fact that the more the *gfp* transcripts were processed by RppH, the more they were cleaved by RNase E, and the more their stem‐loops at the 3′‐terminus were exposed. The *gfp* transcript was swiftly digested by 3′‐to‐5′ exoRNases without a stable stem‐loop, resulting in minimal *gfp* expression. Additionally, the mCherry fluorescence intensity was affected by the stem‐loops, resulting in a significant change in the ratio of GFP to mCherry (Figure 6c). Furthermore, when the RNase E cleavage site was removed, *gfp* expression changed slightly, along with the variation in the stem‐loops in all cases (N_1_ vs N_2_) and was unaffected by RppH processing (Figure 6e). The ratios of GFP/mCherry between distinct stem‐loops ranged from 75‐fold to 1500‐fold (Figure 6c) when the RNase E cleavage site was added, whereas when the RNase E cleavage site was not inserted, this tendency was significantly attenuated (the maximum being only fourfold) (Figure 6f). In summary, the results not only proved the SRPS quantitative regulatory mechanism in *E. coli* but also confirmed the feasibility of using these SRPS elements for synthetic biology applications.

To further explore and utilize SRPS elements, we constructed the first database of prokaryotic SRPS sites (ProSRPSite, http://prosrpsite.sdu.edu.cn/). The ProSRPSite database provides genome‐wide processing sites of various SRPS‐related enzymes from diverse species, such as RNase E in *E. coli* and *S. enterica*; RNase Y in *S. pyogenes*, *B. subtilis*, and *S. aureus*; and 3′‐to‐5′ exoRNases in *E. coli* and *S. pyogenes*. Moreover, the RNA processing sites of unknown enzymes identified using differential RNA‐seq^[^ ^28^ ^]^ in several species were included in the database. The data from ProSRPSite are beneficial for elucidating the mechanisms of SRPS in different species, and their availability should accelerate the application of SRPS in synthetic biology.

## 3 Conclusions

Balanced expression of multiple genes in chassis cells is a central challenge in synthetic biology. Although a polycistronic operon offers the advantages of 1) regulating several genes simultaneously,^[^ ^11^ ^]^ 2) reducing noise in stochastic gene expression,^[^ ^12^ ^]^ 3) facilitating protein complex assembly,^[^ ^13^ ^]^ and 4) minimizing the demand for gene transcriptional resources, it also presents a potential conflict between the equimolar stoichiometry of transcripts within an operon and non‐equimolar stoichiometry of enzymes or subunits that are frequently necessary. One strategy employed by the cell to resolve this conflict is SRPS. Although we understand many of the major processes involved in SRPS,^[^ ^56^ ^]^ we still lack a quantitative predictive understanding of the gene expression stoichiometry, which severely limits the applications of SRPS elements in synthetic biology.

In this study, we profiled the genome‐wide targets of SRPS‐related RppH, RNase E, PNPase, RNase II, and RNase R enzymes in *E. coli*, at single‐nucleotide resolution (Figure 1). For the first time, we used the SEnd‐seq^[^ ^48^ ^]^ method to gain access to the RppH targetome and quantitatively analyze the genome‐wide phosphorylation status of each 5′ end region of the transcript, ’s 5′ terminal region based on the identification of TSSs using SEnd‐seq. The analysis of the transcript 5′ ends generated by RNase E revealed the presence of the consensus sequence ‘RN↓WUU’ (where R is G/A, W is A/U, N is any nucleotide, and ↓ is the site of cleavage) located near the processing sites (Figure 2a), similar to the motif previously proposed for *S. enterica* RNase E.^[^ ^44^ ^]^ The most highly conserved element of this consensus sequence was the U at the two nucleotide positions downstream of the cleavage site (U_+2_), which differed from that of RNase Y, with the preference of a G at position −1 relative to the cleavage site in *S. pyogenes*.^[^ ^45^ ^]^ We then employed an RNA‐seq‐based comparative approach to juxtapose the RNase E‐dependent RNA 3′ ends with 3′‐to‐5′ exoRNase targetomes and discovered that the RNase E‐dependent RNA 3′ ends were mostly derived from PNPase trimming and RNase II nibbling following RNase E processing (Figure 2c). Moreover, there was a significant decrease in the average MFE around the 3′ *rne* end and 3′ *exornase* stop sites (Figure 2b,d). This is consistent with the fact that most of the 3′ ends originating from RNase E cleavage were further trimmed by 3′‐to‐5′ exoRNases whose actions were impeded by a stable stem‐loop, resulting in a low MFE. Most PNPase substrates were fully degraded (Figure 3c), and RNase II had low processivity (3–5 nt) in *E. coli* (Figure 2e). The mechanism of SRPS involves RNase E and three exoRNases; for example, the primary transcripts were sheared by RNase E and further digested by PNPase, RNase II, RNase R, and PNPase (Figure 3g,i). Our study revealed that the majority of targetomes were located in polycistronic operons, especially in the complex‐operon (Table 1; Table S7, Supporting Information). This indicates that SRPS enzymes are involved in modulating the expression ratio of each gene in the complex‐operon.

Although several examples of differential stability within operons have been characterized on a case‐by‐case basis^[^ ^57^ ^]^ and stem‐loops have been shown to modulate the TA ratio of genes encoded on polycistronic mRNAs,^[^ ^16^ ^]^ the mechanism underlying quantitative regulation is yet to be discovered. The quantitative characterization of the SRPS process is critical for understanding the control of stoichiometric gene expression. RppH has a preference for nucleotides on the 5′‐terminus in vitro.^[^ ^50^, ^53^, ^54^ ^]^ However, the quantitative role of RppH in SRPS is unclear, and whether and how it is determined by the nucleotide on the 5′‐terminus remains unclear. To the best of our knowledge, this is the first study to identify a genome‐wide preference for RppH‐processed sites in vivo. We discovered that the first three nucleotides on the 5′‐terminus determined the ratio of transcripts processed by RppH and observed a strictly linear relationship between the processed ratio and sum of scores assigned to the first three nucleotides on the associated transcript (Figure 4a). Furthermore, the stem‐loops associated with the PNPase targetomes exhibited an MFE that was negatively correlated with the termination ratio of PNPase at the 3′‐terminus (Figure 4b). This implies that the stability of the stem‐loop precisely quantifies the termination efficiency of exoRNases, thus determining the TA on a genome‐wide scale. Moreover, we found that 21 RNase E‐mediated 5′‐UTR processing sites in 13 operons resulted in the 5′‐UTR forming different stem‐loops that affected the binding of RBSs to ribosomes, and thus, modulated translation efficiency (Figure 5). The 5′‐UTR processing‐mediated translational regulation of ribosomal and thermosomal genes has also been discovered in *Methanolobus psychrophilus*, although the enzyme involved is yet to be identified.^[^ ^58^ ^]^ Even though RNase III‐mediated 5′‐UTR processing has been reported in *E. coli* for individual genes,^[^ ^59^, ^60^ ^]^ this is the first time that the regulation of translation efficiency by RNase E‐mediated 5′‐UTR processing has been identified genome‐wide in bacteria. This mechanism achieves optimal translation efficiency and facilitates adaptation to environmental changes through condition‐specific RNA processing.^[^ ^61^ ^]^

Here, we constructed an operon consisting of a promoter and two different fluorescent genes, to assess the function of the identified SRPS elements in optimizing gene expression in an operon. First, we found that the first two nucleotides of the TSS were essential for modulating the expression ratio of the first gene (*gfp*) carried by different stem‐loops (Figure 6b,d). Second, the stable stem‐loop significantly enhanced *gfp* expression and the ratio of GFP to mCherry (Figure 6b,c). Third, RNase E cleavage is necessary to release the stem‐loop and thereby regulate the stoichiometry of the *gfp* and *mcherry* transcripts (Figure 6b,e). These discoveries allowed us to understand the mechanism by which gene expression is modulated in *E. coli*. In addition, these highlighted the importance of the identified synthetic biology elements for the optimization of biosynthetic pathways to generate desired products and gain the highest returns.

The SRPS mechanism has the following characteristics: Compared to other regulatory mechanisms, such as engineered promoters,^[^ ^62^ ^]^ protein degradation,^[^ ^63^ ^]^ CRISPRi,^[^ ^64^ ^]^ and RNA degradation‐tuning sequences or structural determinants,^[^ ^65^, ^66^, ^67^ ^]^ the regulatory SRPS mechanism is multi‐layered, as it determines the TA and modulates translation efficiency. In the post‐transcriptional layer, RNA processing and degradation mediated by the SRPS mechanism can redistribute ribosomes (a key restrictive resource in *E. coli* ^[^ ^61^ ^]^) more quickly and efficiently. Studies have shown that programmed RNA decay can modulate synthetic circuit resource allocation.^[^ ^68^ ^]^ Moreover, translation is one of the most energy‐consuming processes in any cell (related processes account for more than 40% of the cell's energy budget) and different genes in the same transcript can have translation efficiencies that differ by up to 100‐fold.^[^ ^69^ ^]^ The translational initiation rate modulated by SRPS, rather than the elongation rate, is regarded as a determinant of translation efficiency.^[^ ^70^ ^]^

Second, the SRPS can be regulated quantitatively. The processing efficiency of RppH is determined by the first three RNA nucleotides at the 5′‐terminus of the primary transcript, and in turn determines the cleavage efficiency of endoRNases by the cascade. After the transcript is cleaved by the endonuclease, there is a significant negative correlation between the free energy of folding (Δ*G*) of the stem‐loops formed at the 3′ end of the RNA fragment and the degradation termination rate of the exonuclease in the stem‐loop (Figure 4b). The smaller the Δ*G* is, the more stable the stem‐loop and the higher the degradation termination efficiency. Conversely, the larger the Δ*G* is, the more unstable the stem‐loop and the lower the degradation termination efficiency. Thus, the TA of the upstream genes can be quantitatively adjusted based on the degree of stability of the stem‐loop. Moreover, after the endoRNase digestion site of the 5′‐UTR of the transcript is cleaved by an endonuclease, the secondary structure formed at the 5′ end of the downstream RNA fragment affects the exposure of RBS elements and quantitatively determines the translation efficiency of the transcript by affecting the affinity between the RBSs and ribosomes. Finally, by fine‐tuning these multi‐layers, the gene expression stoichiometry was quantitatively modulated.

Third, SRPS is ubiquitous in bacteria. Ribonuclease phylogenetic analysis indicated that almost all bacteria contained endoRNases, exoRNases, and oligoribonucleases.^[^ ^71^ ^]^ Several studies have shown that the SRPS mechanism is widespread in the model gram‐negative bacterium *E. coli* ^[^ ^72^ ^]^ and gram‐positive bacteria *C. cellulolyticum* ^[^ ^16^ ^]^ and *B. subtilis*.^[^ ^27^ ^]^ The SRPS thus appears to be a common strategy to allow deviation from one‐to‐one stoichiometry for co‐transcribed genes in the polycistronic operon, despite differences in the mRNA processing enzymes.

In conclusion, we present the first single‐nucleotide resolution genome‐wide targetomes of SRPS‐related enzymes in *E. coli*, revealing the interplay between a pyrophosphohydrolase, an endoRNase, and exoRNases, and portraying the stoichiometric regulatory model of SRPS in *E. coli*. A dual‐fluorescence reporter system was used to verify the effectiveness of SRPS elements. Finally, we constructed the first prokaryotic SRPS site database (ProSRPSite), which not only provides genome‐wide SRPS sites of various enzymes in diverse species but also lays a solid foundation for the application of SRPS in synthetic biology. Our discovery presents a multi‐layer, quantitative, and ubiquitous post‐transcriptional regulatory mechanism as well as elements to accurately optimize gene expression stoichiometry. In addition, it provides a solution for generating “polycistronic operon plus SRPS elements”, to overcome the classic challenge faced by synthetic biology researchers, that is, “balanced expression of multiple genes in chassis cells”.

## 4 Experimental Section

### Strains and Culture Conditions

The strains and plasmids used in this study are listed in Table S9 (Supporting Information). The strains were cultured in Luria Bertani medium at 37 °C, and antibiotics were added to the medium in appropriate amounts, as per requirement. The concentrations of kanamycin (Kan), spectinomycin, and ampicillin used were 50, 50, and 100 µg mL^−1^, respectively.

### Construction of Mutants

Mutants of Δ*pnp* from *E. coli* MG1655 were generated using the Wanner method.^[^ ^73^ ^]^ The primers and plasmids used are listed in Tables S9 and S11 (Supporting Information), respectively. Approximately 60‐bp regions upstream and downstream of the *pnp* gene were ligated with the Kan resistance gene by means of PCR, using pCL1920 as the template and the primers pcl1920‐*pnp*‐F/R. The PCR product thus obtained was electrotransformed into MG1655 cells. The transformants were selected based on their Kan resistance and verified by means of PCR and sequencing. For complementation, the *pnp* genes were cloned into linearized pCL1920 using an In‐Fusion HD Cloning Kit (TaKaRa, Beijing, China). The correct plasmid was then transferred into the corresponding deletion strain.

### Selection and Analysis of SEnd‐ and RNA‐seq Results

The list of select SEnd‐ and RNA‐seq results is shown in Table S10 (Supporting Information). The data were subjected to quality control analysis using FastQC (v0.11.8). Adapter sequences were removed using Trimmomatic software (v0.39). Paired end reads of SEnd‐seq were merged into single end reads using FLASH (v1.2.11). The correlated 5′ and 3′ end sequences were extracted as previously described in the study.^[^ ^48^ ^]^ Subsequently, the processed paired end reads of SEnd‐seq were extracted from the total library by searching for the special 5′‐adapter ‘AAACTCTCCACGTNNNN’. The total and processed paired end reads of SEnd‐seq were mapped to the reference *E. coli* genome NC_000913.3 using Hisat2 (v2.1.0). Only paired end reads that were uniquely mapped to the same strand and had an insert length of <10000 nt were used for further analysis. The paired end RNA‐seq reads were mapped to the same reference genome. Gene expression was determined using FeatureCounts (v1.6.4) and the FPKM for each gene was calculated using the following formula:

(1) $$\mathrm{FPKM}\;=\;\frac{\mathrm{Unique}\;\mathrm{mapped}\;\mathrm{fragments}\;\mathrm{of}\;\mathrm{gene}\;\times \;10^{9}}{\mathrm{Total}\;\mathrm{unique}\;\mathrm{mapped}\;\mathrm{fragments}\;\times \;\mathrm{length}\;\mathrm{of}\;\mathrm{gene}}$$

Finally, the average FPKM for each gene calculated from all replications was used for further analysis.

### Identification of Processed Sites Associated with SRPS Enzymes RppH

Three processed libraries were extracted from the total library, based on the presence of the special 5′‐adapter sequence. Briefly, the processed RNA (5′‐P_i_ RNA) was ligated to a special 5′‐adapter using T4 RNA ligase I (New England Biolabs, Beijing, China), while the primary RNA (5′‐PPP_i_ RNA) was not. A custom shell script was then used to search for adaptor‐labeled reads in the total library, thereby specifically extracting processed RNA as a processed library. The parameters for identifying the processing positions of RppH, which refers to the method for identifying the TSS using SEnd‐seq data,^[^ ^48^ ^]^ were as follows: in the alignment results of processed libraries, 1) only positions with >3 reads starting at this position, with an increase of at least 30% in read coverage from its upstream to its downstream were retained; 2) Candidate processing positions within five bases in the same orientation were clustered together, and the position with the largest number of reads was used as the representative processing position; 3) The positions were retrieved from at least two samples; 4) Only processing positions located within 5 nt of the identified TSS^[^ ^48^ ^]^ were defined as the RppH processing sites, while the others were determined to be processed by other enzymes (see details in Figure S1a, Supporting Information). The wobble of transcription initiation was considered when identifying RppH processing sites. A method was referred to that identified the TSS using SEnd‐seq data, to detect the processing site of RppH. First, the wobble of transcription initiation may lead to an increase in the coverage of a total of 5 nt sites flanking the exact TSS site; therefore, bins of +/‐2 nt were taken as candidate processing positions (5 nt). If the bin meets the filtering criteria (i.e., read start number of each position in bin ≥10 and bin+1 coverage ≥1.3 × bin coverage), then the position with the highest number of reads starting in the locus (5 nt) was identified as the final processing position. As a result, this site was the most reliable for minimizing the influence of the wobble on transcription initiation. If the final position was located within 5 nt of the identified TSS, the final position belonged to RppH; otherwise, it belonged to other enzymatic processes.

### RNase E

To identify the RNase E processing positions, the previously reported methods were used.^[^ ^45^, ^47^, ^49^ ^]^ First, only 5′ or 3′ ends of transcripts with a count per million value ≥3 in total libraries of two replications for Δ*rne* or three replications for WT were further analyzed. To account for compositional biases, the end‐counts were normalized using the trimmed mean of the M‐value normalization. The differentially expressed ends were then defined using edgeR (v3.28.1), while end‐counts with a log2‐fold change (log_2_FC, WT vs Δ*rne*) ≥1 and false discovery rate <0.05 were counted. Additionally, these positions were further filtered using two parameters: the proportion of ends at a position in the WT strain was 1) counted as the number of reads starting or ending at this position divided by the coverage at this position, which was set to ≥2%; and 2) threefold the proportion of ends in Δ*rne*. Finally, positions that overlapped with those processed by the other enzymes described above were identified as the 5′ *rne* end, while there was no more restriction for the 3′ *rne* end. When at least two consecutive positions were identified within a window of 5 nt, the position with the highest proportion of ends in the WT was chosen for further analysis (see detail in Figure S1b, Supporting Information).

### PNPase, RNase II, and RNase R

The libraries of Δ*pnp*, Δ*rnb*, and Δ*rnr* were one replication. The first step to identifying the processing positions of these 3′‐to‐5′ exoRNases was same as described above for finding the RNase E processing site. The differentially expressed ends were then defined using edgeR (v3.28.1), based on the criteria: log_2_FC ≥1 or ≤−1 and false discovery rate <0.05. Subsequently, the positions with a proportion of ends in the WT of ≥2% and a ratio between the WT and Δ*exornase* proportions of ends of ≥3, or positions with a proportion of ends in the Δ*exornase* of ≥2% and a ratio between the Δ*exornase* and WT proportions of ends of ≥3 were further analyzed. Finally, the 3′ ends of the transcripts that were more abundant in the WT were identified as 3′ *exornase* stops. The 3′ and 5′ ends that were more abundant in the Δ*exornase* strain were identified as the 3′ and 5′ *exornase* start sites, respectively. Similarly, when there were at least two positions in a window of 5 nt, the position with the highest proportion of ends in the reference strain was used for further analyses (see detail in Figure S1c, Supporting Information).

### Analysis of Nucleotides and Structures Around the Processing Sites

Nucleotide motifs surrounding the processing positions of SRPS enzymes were generated using WebLogo (v3.6.0). RNAfold (v2.4.14) was used to calculate the MFE (Δ*G*/kcal·mol^–1^) using a sliding window of 50 nt, with a 100 nt center at the processed positions. The average MFE was calculated for each position in the 100 nt window around the processed positions.

### Comparison of the RNase E and Major exoRNase Processing Sites in *E. coli*

The processing positions of RNase E were compared with those of the exoRNases. First, the 3′ *rne* end was compared with the 3′ *exornase* stop (trimming stop positions of exoRNases), which is located less than 5 nt downstream or upstream of the 3′ *rne* end. Second, the 3′ *rne* end, which corresponds to the 3′ *exornase* stop, was compared with the 3′ *exornase* start (trimming start positions of exoRNases) located downstream. The maximum distance between the trimming stop and start positions was set to 300 nt for PNPase and RNase R, and 10 nt for RNase II. Third, the 3′ *rne* end was compared with the 3′ *exornase* start, allowing a ±5 nt shift. Finally, the 5′ *rne* end was compared with the 3′ *exornase* start located less than 10 nt upstream. Additionally, two fragments generated by RNase E were detected (the 5′ *rne* end was located less than 300 nt upstream of the 3′ *rne* end). These comparisons were performed using AWK software (v4.0.2).

### Analysis of the Associations Between the Processing Ratio and the First Three Sequences of TSSs Processed by RppH

The processing ratio of the RppH processing sites was calculated using the following formula:

(2) $$\mathrm{Processed}\;\mathrm{ratio}\;=\frac{\mathrm{Sum}\;\mathrm{of}\;\mathrm{processed}\;5^{\prime }\;\mathrm{ends}\;\mathrm{around}\;\mathrm{processed}\;\mathrm{site}\;}{\mathrm{Sum}\;\mathrm{of}\;\mathrm{total}\;5^{\prime }\;\mathrm{ends}\;\mathrm{around}\;\mathrm{processed}\;\mathrm{sites}}$$

The sum of the processed 5′ ends around the processed positions was represented as the sum of the 5′ end‐counts, which were retrieved from the alignment results of the processed paired end reads from 4 nt upstream to 4 nt downstream of the processed positions. The sum of the 5′ ends around the processed sites was calculated in the same manner; the only difference was that the 5′ end‐counts were retrieved from the alignment results of the total paired end reads. The average processing ratios of all the replicates for each position were calculated. Finally, the average processing ratio of the processed positions was calculated with the same first three nucleotides, and the correlations between these processing ratios and the sum of the scores of the first three sequences. Plots were generated using OriginPro 2020 software (OriginLab, Massachusetts, USA).

### Calculation of the End Ratio of 42 PNPase Trimming Stop Sites and the MFE of Sequences Located Upstream of these Positions

42 3′ pnp stop sites that paired with 3′ pnp start were selected, to calculate the end ratios of these positions using the following formula:

(3) $$\mathrm{End}\;\mathrm{ratio}\;=\;\frac{3^{\prime }\;\mathrm{ends}\;\mathrm{of}\;\mathrm{transcripts}\;\mathrm{around}\;3^{\prime }\;\mathrm{pnp}\;\mathrm{stop}}{3^{\prime }\;\mathrm{ends}\;\mathrm{of}\;\mathrm{transcripts}\;\mathrm{from}\;3^{\prime }\;\mathrm{pnp}\;\mathrm{stop}\;\mathrm{to}\;3^{\prime }\;\mathrm{pnp}\;\mathrm{start}}$$

The total 3′ ends of the transcripts were counted as the sum of the 3′ ends 5 nt upstream of the 3′ *pnp* stop site to 5 nt downstream of the 3′ *pnp* start site that was paired with the 3′ *pnp* stop site. The 3′ ends of the transcripts around the 3′ *pnp* stop site were counted as the sum of the 3′ ends of the 11 nt centered on the 3′ *pnp* stop site. The average MFE of the sequences was then predicted located 50–100 nt upstream of the 3′ *pnp* stop site. Plots were generated using OriginPro 2020 software.

### Identification of Complex‐Operon

The FPKM of each gene was calculated as described above. If the FPKM values of two adjacent genes within the same operon were both ≥2 and their ratio was ≥3 or ≥1/3, or if one of them was <2 and the absolute value of their difference was ≥10, the operon was determined to be a complex‐operon exhibiting a highly skewed TA landscape.

### Northern Blot Assays

Cultures of MG1655 and its mutants at log‐phase, with OD_600_ values of 2.5–3, were collected and total RNA was extracted from them using the TaKaRa MiniBEST Universal RNA Extraction Kit (TaKaRa, Beijing, China). Total RNA was separated on 1% agarose‐formaldehyde gels and transferred onto nylon membranes (Hybond N+; GE Healthcare, Chicago, USA) using a Bio‐Rad vacuum blotter (Bio‐Rad, California, USA). RNA was UV‐crosslinked to the membranes and DIG‐labeled RNA probes were generated for detection, using the DIG Northern Starter Kit (Roche, Basel, Switzerland). Hybridization was performed overnight at 68 °C, in Easy Hyb buffer. Target bands were visualized by means of chemiluminescence using CDP‐Star. A RiboRuler High Range RNA Ladder (Thermo Scientific, Shanghai, China) was used to assess the size of the RNA transcripts and 5S rRNA was used as a loading control. Each northern blot was performed in triplicates.

### qRT‐PCR Analysis

Cells at log‐phase were harvested by means of centrifugation at 10000×*g*, 4 °C for 10 min. Total RNA was isolated using the TaKaRa MiniBEST Universal RNA Extraction Kit (TaKaRa, Beijing, China), and the RNA concentration was verified using Qubit 4 (Thermo Fisher, Waltham, USA). cDNA was synthesized using the PrimeScript RT Reagent Kit with gDNA Eraser (TaKaRa, Beijing, China). A SYBR Premix Ex Taq II Kit (TaKaRa, Beijing, China) was used for qRT‐PCR and the reactions were run on a LightCycler 480 II Sequence Detection System (Roche, Basel, Switzerland). The primers used to amplify the target genes are listed in Table S11 (Supporting Information), and *gapA* was used as the reference gene. The results were analyzed using the 2^−ΔΔCT^ method.

### Identification of Operons Cleaved by RNase E at least Twice in the 5′‐UTR or in the Same IGR

The operons satisfying the following criteria were retrieved: First, the operon that covered at least one 5′ *rne* end in the 5′‐UTR and located less than 200 nt downstream of the TSS. Alternatively, the operon that covered at least two 5′ *rne* ends in the same IGR, and located within a distance of <200 nt between adjacent 5′ *rne* ends. Second, all of these transcripts with different 5′‐UTR lengths were required to exist in the total RNA library.

### RACE

Total RNA (2 µg) was used to synthesize first‐strand cDNA with the cDNA synthesis primer SP1, using Transcriptor Reverse Transcriptase (Roche, Basel, Switzerland). The product was purified using the High Pure PCR Product Purification Kit (Roche, Basel, Switzerland). A homopolymeric A‐tail was added to the 3′ end of the first‐strand cDNA using recombinant terminal transferase and dATP. Nested PCR was performed using the cDNA as a template, to obtain gene‐specific products. The PCR products were cloned into the pMD19‐T vector (TaKaRa, Beijing, China) for further sequencing.

### Functional Analysis of the 5′‐UTR In Vitro

To assess the effect of the 5′‐UTR on the translation efficiency of downstream genes, an mCherry reporter system was constructed (Figure S7, Supporting Information). The mCherry reporter system included *lac* promoter, TSS, 5′‐UTR, and mCherry. The TSS was verified using 5′ RACE. The 5′‐UTRs of *bcp*, *eno*, and *srlD* were fused with *mcherry*, cloned into pBBR1MCS‐2, and transformed into the RNase E inactivation strain. pBBR1MCS‐2 is a low‐copy number plasmid with a *lac* promoter and T7 terminator. The fluorescence intensity of mCherry was detected at excitation and emission wavelengths of 580 and 610 nm, respectively, using a Synergy H1 microplate reader (Biotek, Vermont, USA). The final fluorescence intensity was normalized to the OD_600_ value.

### Functional Analysis of Stem‐Loops and RNase E Cleavage Sites In Vitro

A dual‐fluorescence reporter system was constructed to evaluate the effects of the stem‐loop and RNase E cleavage sites. The *gfp* (encoding green fluorescent protein) and *mcherry* (encoding red fluorescent protein) genes were expressed in a single operon with a *lacI* promoter. Different stem‐loops harboring intergenic regions were introduced into the site between the two genes. The RNase E cleavage site was located between the stem‐loops and *mcherry*, to assess the influence of RNase E cleavage. The fluorescence intensity of mCherry was detected at excitation and emission wavelengths of 580 and 610 nm, respectively, while that of GFP was detected at excitation and emission wavelengths of 482 and 515 nm, respectively.

Analysis of the Correlation Between the Processing Ratio and First Three Nucleotide Sequences of the Transcript In Vitro

The first and second A bases after the TSS of the lacI promoter in the plasmid pBR2‐gfp‐loop1/2(+/−)‐mCherry were mutated to C, G, or T using the QuikChange site‐directed mutagenesis method,^[^ ^74^ ^]^ which resulted in eight TSSs with different scores (CCT = 3, CAT = 7, GAT = 8, ACT = 10, TAT = 10, ATT = 13, AAT = 14, and AGT = 14). The primers used for this are listed in Table S11 (Supporting Information).

### Statistics Analysis

The measurements were performed in triplicate and independently repeated at least three times, except otherwise described. Data were given as mean ± standard deviation (s.d.). Statistical analysis was performed with GraphPad Prism 8.0 software (GraphPad, San Diego, USA). Two‐tailed Student's t‐test was applied to detect differences between the two groups with a single independent factor. P < 0.05 was considered statistically significant.

### Accession Numbers

The SEnd‐ and standard RNA‐seq datasets from this study can be found in the National Center for Biotechnology Information Gene Expression Omnibus, under Accession Number GSE117737.

## Conflict of Interest

The authors declare no conflict of interest.

## Author Contributions

D.L., H.L., and Y.W. contributed equally to this work. R.H. designed research; D.L. and J.C. performed experiments; H.L. and R.H. analyzed data; D.L. constructed the database; R.H., H.L., and D.L. wrote the paper.

## Supporting information

Supporting Information

Supporting Table 1–2

Supporting Table 3

Supporting Table 4

Supporting Table 5

Supporting Table 6

Supporting Table 7‐8

## Data Availability

The data that support the findings of this study are openly available in Gene Expression Omnibus (GEO) at https://www.ncbi.nlm.nih.gov/geo/query/acc.cgi?acc=GSE117737, reference number 117737. These data were derived from the following resources available in the public domain: [SRS3599271], [https://sra‐downloadb.be‐md.ncbi.nlm.nih.gov/sos3/sra‐pub‐zq‐22/SRR007/609/SRR7609049.sralite.1]; [SRS3599272], [https://sra‐downloadb.be‐md.ncbi.nlm.nih.gov/sos5/sra‐pub‐zq‐14/SRR007/609/SRR7609050.sralite.1]; [SRS4257704], [https://sra‐downloadb.be‐md.ncbi.nlm.nih.gov/sos5/sra‐pub‐zq‐16/SRR008/449/SRR8449221.sralite.1]; [SRS4257705], [https://sra‐downloadb.be‐md.ncbi.nlm.nih.gov/sos5/sra‐pub‐zq‐16/SRR008/449/SRR8449222.sralite.1]; [SRS4257706], [https://sra‐downloadb.be‐md.ncbi.nlm.nih.gov/sos3/sra‐pub‐zq‐22/SRR008/449/SRR8449223.sralite.1]; [SRS4257708], [https://sra‐downloadb.be‐md.ncbi.nlm.nih.gov/sos5/sra‐pub‐zq‐16/SRR008/449/SRR8449225.sralite.1]; [SRS4257710], [https://sra‐downloadb.be‐md.ncbi.nlm.nih.gov/sos3/sra‐pub‐zq‐22/SRR008/449/SRR8449227.sralite.1]; [SRS4257712], [https://sra‐downloadb.be‐md.ncbi.nlm.nih.gov/sos5/sra‐pub‐zq‐16/SRR008/449/SRR8449229.sralite.1]; [SRS4257718], [https://sra‐downloadb.be‐md.ncbi.nlm.nih.gov/sos3/sra‐pub‐zq‐22/SRR008/449/SRR8449235.sralite.1]; [SRS4257719], [https://sra‐downloadb.be‐md.ncbi.nlm.nih.gov/sos5/sra‐pub‐zq‐16/SRR008/449/SRR8449236.sralite.1]; [SRS4257720], [https://sra‐downloadb.be‐md.ncbi.nlm.nih.gov/sos5/sra‐pub‐zq‐16/SRR008/449/SRR8449237.sralite.1].
