# Supplementary material for: Selective RNA Processing and Stabilization are Multi‐Layer and Stoichiometric Regulators of Gene Expression in Escherichia coli
Source: Adv Sci (Weinh). 2023 Oct 16;10(33):2301459. doi: 10.1002/advs.202301459 (PMC10667835; doi:10.1002/advs.202301459)
Supplement: Supplementary file 1 — Supporting Information [file ADVS-10-2301459-s001.pdf]

ADVANCED SCIENCE
Open Access

Supporting Information

for *Adv. Sci.*, DOI 10.1002/advs.202301459

Selective RNA Processing and Stabilization are Multi-Layer and Stoichiometric Regulators of Gene Expression in *Escherichia coli*

*Daixi Liu, Haibo Lv, Yafei Wang, Jinyu Chen, Dexin Li and Ranran Huang**

Supplementary Material

Selective RNA processing and stabilization are a multi-layer and stoichiometric regulation of gene expression in *Escherichia coli*

Daixi Liu[1,2*], Haibo Lv[1*], Jinyu Chen[1*], Yafei Wang[1], Dexin Li[3], Ranran Huang[1§]

[1]Institute of Marine Science and Technology, Shandong University, 72 Binhai Road, Qingdao, 266237, China

[2]School Of Pharmaceutical Sciences, Shandong University, 44 Wenhuaxi Road, Jinan, Shandong, 250012, China

[3]School of Computer Science and Technology, Shandong University, Qingdao, Shandong, 266237, China

[*] These authors contributed equally to this work as co-first authors

[§] To whom correspondence should be addressed. Tel: +86 15092288609; Email: huangrr@sdu.edu.cn

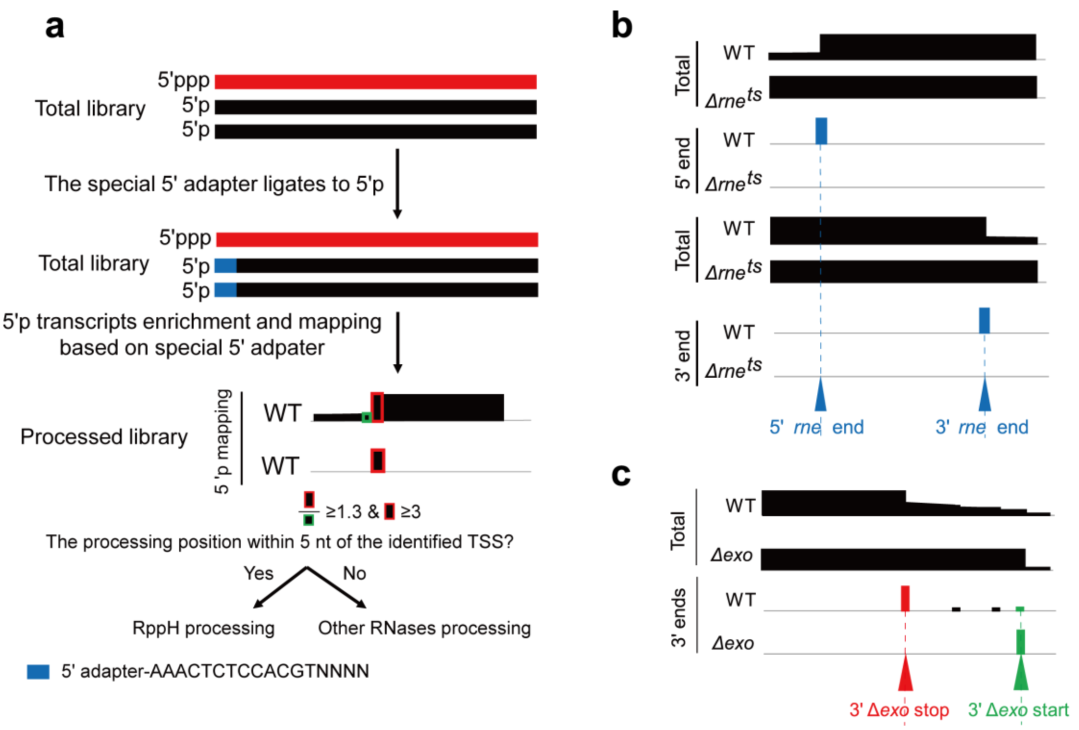

Supplementary Figure 1. The methods of identifying the processing positions of RppH, RNase E and three exoRNases. (a) The processing transcripts were enriched from total library based on the special 5′ adapter, then were mapped on the reference genome. Only positions with more than 3 reads starting and with an increase of at least 30% in read coverage from its upstream to its downstream were retained. Then the positions within 5 nt of the identified TSS were defined as RppH processing positions, while the others were processed by other RNases. (b) The transcript was cleaved by RNase E in wild type (WT) while as full length in *rne*-3071(ts) mutant. The transcript 5′ or 3′ end, which was more abundant in WT than in *rne*-3071(ts) mutant, will be identified as 5′ or 3′ *rne* end, respectively. (c) Schematic representation of RNA sequencing profiling with annotated 3′-to-5′ exoRNases trimming start (more abundant in the Δ*exo*) and trimming stop (more abundant in the WT).

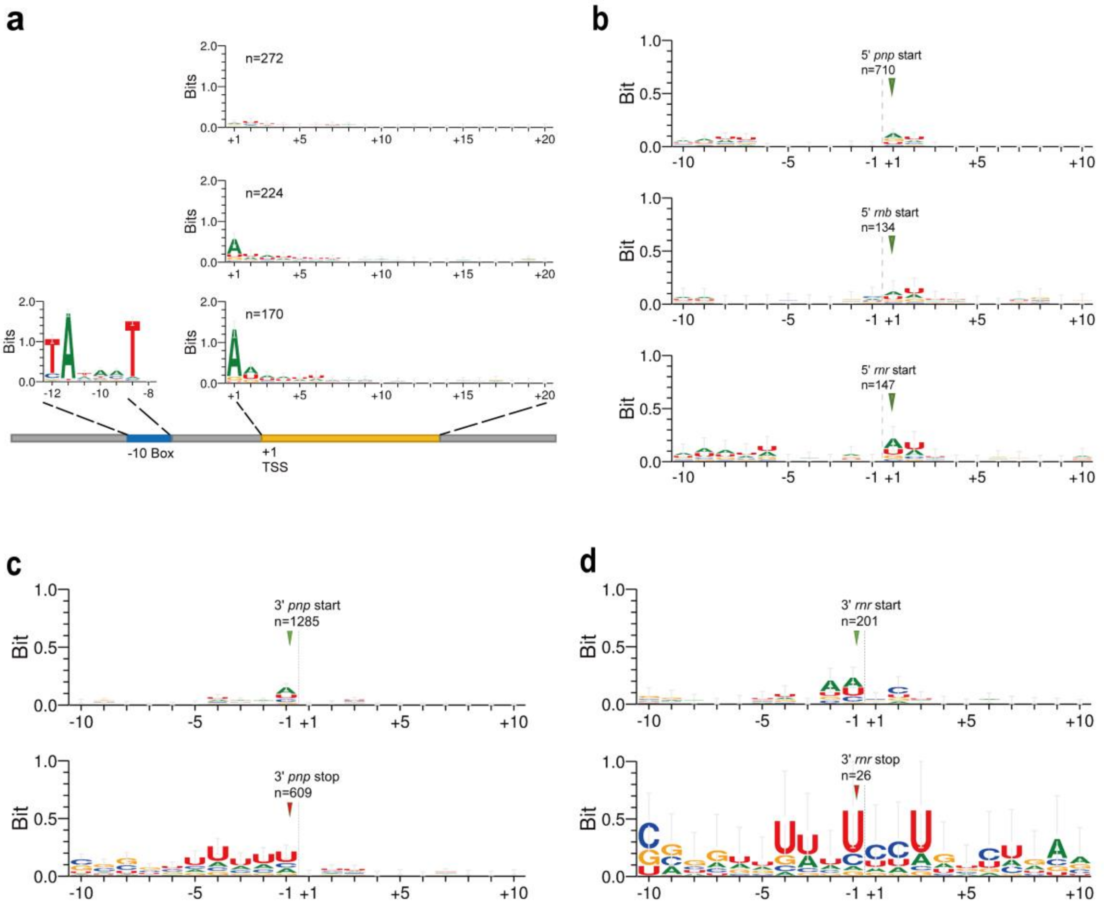

Supplementary Figure 2. Sequence conservation of targetomes of RppH and three exoRNases. (a)The left panel: Motif of -10 element from all processed sites of RppH. The right panel: Motif of processed sites of RppH with processed ratio less than 10% (upper), equal to or greater than 10% and less than 20% (middle), and equal to or greater than 20% (bottom). (b, c and d) Sequence conservation of 5′ Δ*exo* start (b), 3′ *pnp* start/stop (c), and 3′ *rnr* start/stop (d). The sequence logos are based on the sequence alignment of each position surrounding the processed sites.

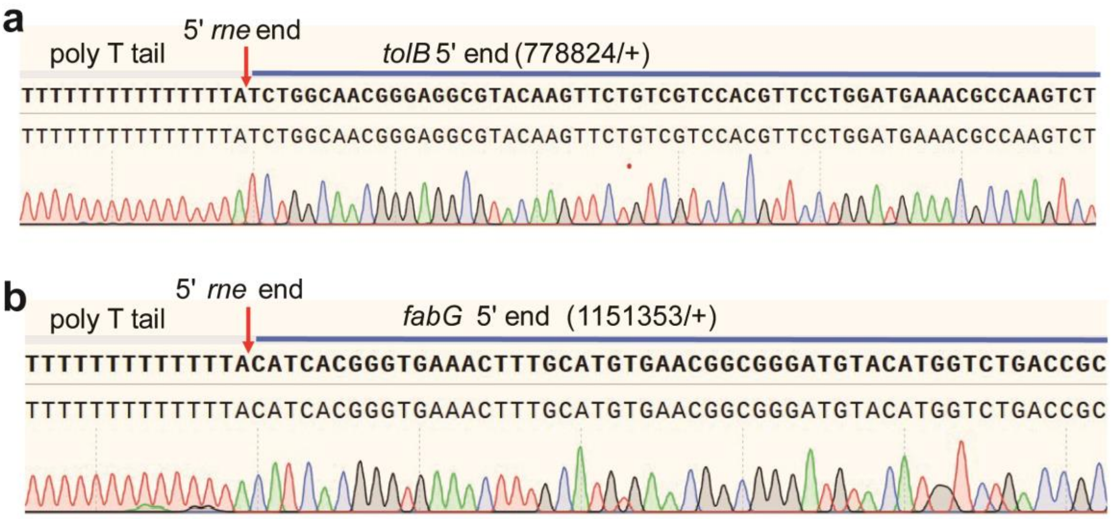

Supplementary Figure 3. 5′ *rne* end were confirmed by 5′ RACE. Positions of 778824 on sense strand (a), and 1151353 on sense strand (b) where cleaved by RNase E was confirmed by 5′ RACE.

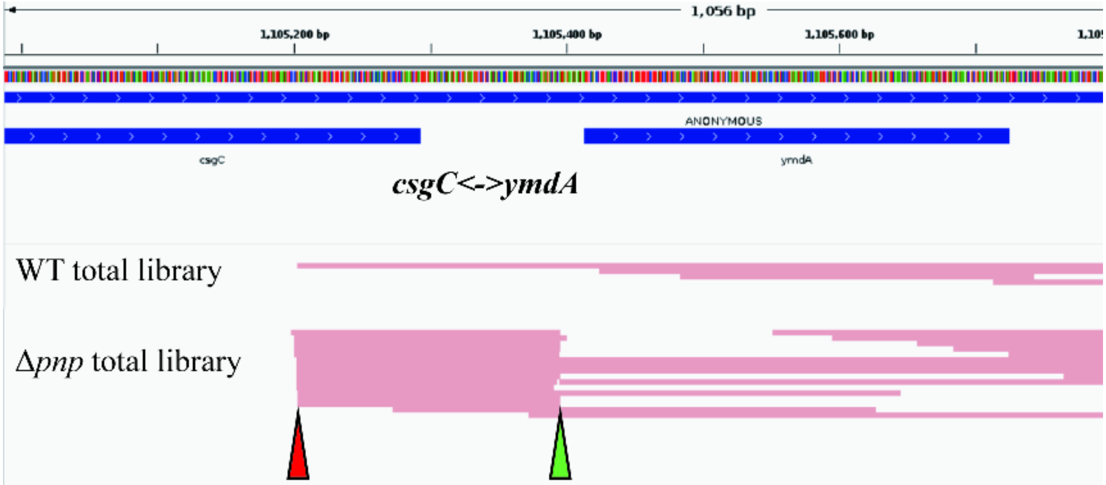

Supplementary Figure 4. The real reads coverage of the fragment in Fig. 3B were visualized with the IGV.

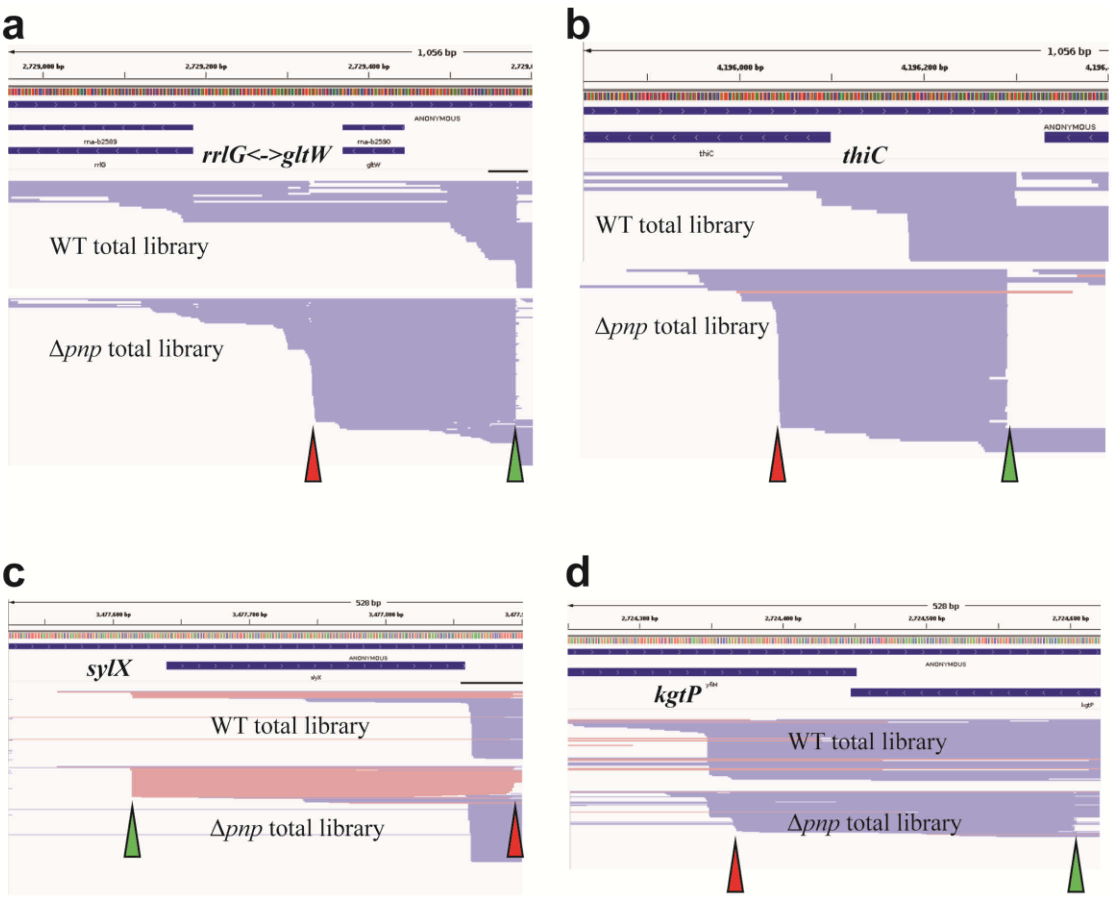

Supplementary Figure 5. The four fragments confirmed by northern blot shown in IGV. The red and green triangles point to the 3′ and 5′ end of four fragments which fully degraded by PNPase, respectively. (a) *rrlG<->gltW*; (b) *thiC*; (c) *sylX*; (d) *kgtP*.

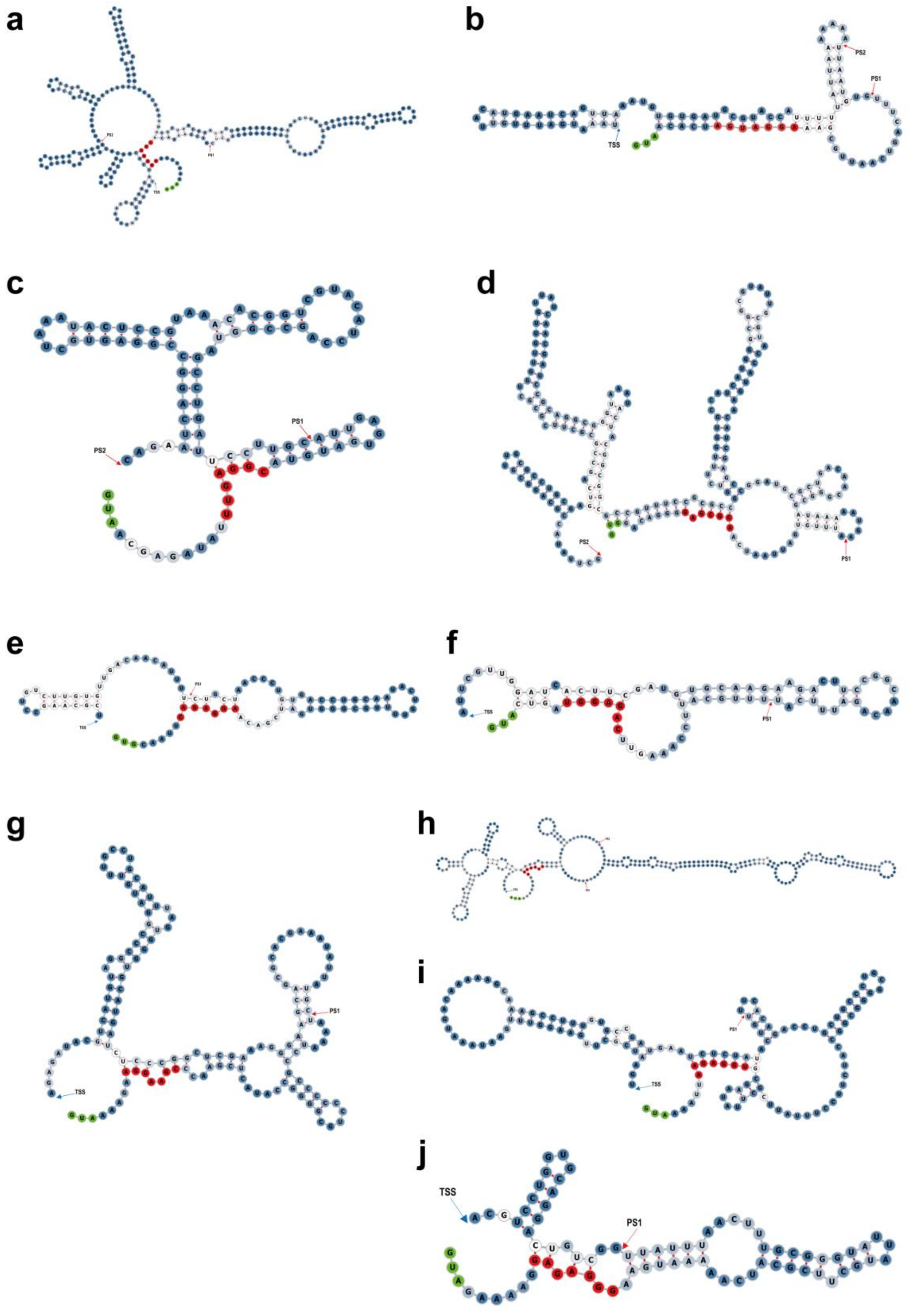

Supplementary Figure 6. The stem-loops formed by 5′-UTR of various transcripts cleaved by RNase E. Schematic representation of the stem-loops formed by 5′-UTR of *ahpC* (a), *elbB* (b), *iscS* (c), *murE* (d), *pepD* (e), *pfkA* (f), *ribE* (g), *tnaA* (h), *tpiA* (i) and *yajQ* (j), respectively.

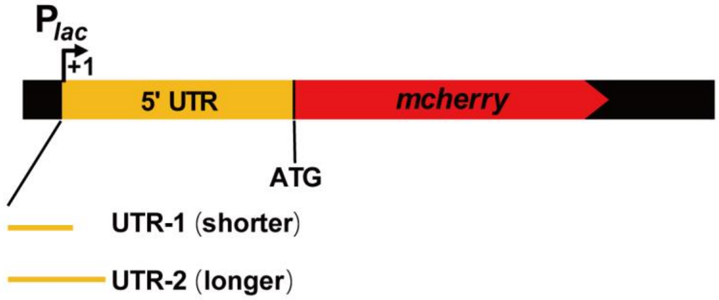

Supplementary Figure 7. The mCherry reporter system with different 5′-UTR. A schematic depicts the mCherry reporter system inserting two different 5′-UTRs of *eno*, *bcp* and *srlD*, respectively.

Supplementary Table 1 . RppH processed sites identified by our method (separate excel file).

Supplementary Table 2. Processed sites by other enzymes identified by our method (separate excel file).

Supplementary Table 3. Processed sites of RNase E, PNPase, RNase II and RNase R identified by our method (separate excel file).

Supplementary Table 4. List of transcripts partially degraded by exoRNases (separate excel file).

Supplementary Table 5. List of transcript fragments accumulating in Δexornases (separate excel file).

Supplementary Table 6. List of transcripts cleaved by RNase E and subsequently degraded by one or more exoRNases (separate excel file).

Supplementary Table 7. The processing positions of SRPS-enzymes inside each operon (separate excel file).

Supplementary Table 8. List of transcripts with at least two different 5′-UTR caused by RNase E (separate excel file).

Supplementary Table 9. Strains and plasmids used in this study

Strain or plasmid	Description/characteristic	Source/reference
**Strains**		
**MG1655**	*Escherichia coli* strain MG1655	This study
**MG1655Δ*pnp***	MG1655 with *pnp* deletion	This study
**MG1655Δ*pnp::pnp***	MG1655Δ*pnp* with pCL1920-*pnp*	This study
***E. coli* DH5α**	Cloning strain	Novagen
***E. coli* BL21(DE3)**	Cloning strain	Novagen

**Plasmids**		
**pBBR1MCS-2***	expression vector	This study
**pJET1.2-Blunt**	Cloning vector	Thermo Fisher
**pCL1920**	expression vector	This study
**pCL1920-*pnp***	pCL1920 containing *E. coli pnp*	This study
**pBR2-*srlD1-mcherry***	pBR2 containing 5′-UTR-1 of *srlD* and *mcherry*	This study
**pBR2-*srlD2-mcherry***	pBR2 containing 5′-UTR-2 of *srlD* and *mcherry*	This study
**pBR2-*eno1-mcherry***	pBR2 containing 5′-UTR-1 of *eno* and *mcherry*	This study
**pBR2-*eno2-mcherry***	pBR2 containing 5′-UTR-2 of *eno* and *mcherry*	This study
**pBR2-*bcp1-mcherry***	pBR2 containing 5′-UTR-1 of *bcp* and *mcherry*	This study
**pBR2-*bcp2-mcherry***	pBR2 containing 5′-UTR-2 of *bcp* and *mcherry*	This study
**pBR2-*gfp-mcherry***	pBR2 containing *gfp* and *mcherry*	This study
**pBR2-*gfp*-loop$_1$(+)-*mcherry***	pBR2 containing *gfp*, stem-loop with ΔG of -25.4 kcal·mol^{-1} , RNase E cleavage site and *mcherry*	This study
**pBR2-*gfp*-loop$_1$(-)-*mcherry***	pBR2 containing *gfp*, stem-loop with ΔG of -25.4 kcal·mol^{-1} and *mcherry*	This study
**pBR2-*gfp*-loop$_2$(+)-*mcherry***	pBR2 containing *gfp*, stem-loop with ΔG of -9.3 kcal·mol^{-1}, RNase E site and *mcherry*	This study
**pBR2-*gfp*-loop$_2$(-)-*mcherry***	pBR2 containing *gfp*, stem-loop with ΔG of -9.3 kcal·mol^{-1} and *mcherry*	This study
**pBR2-*gfp*-loop$_1$(+)-*mcherry* (CAT)**	pBR2-gfp- loop$_1$(+)-*mcherry* with the first A of TSS mutating to C	This study
**pBR2-*gfp*-loop$_1$(-)-*mcherry* (CAT)**	pBR2-gfp-loop$_1$(-)-*mcherry* with the first A of TSS mutating to C	This study
**pBR2-*gfp*-loop$_1$(+)-*mcherry* (TAT)**	pBR2-gfp- loop$_1$(+)-*mcherry* with the first A of TSS mutating to T	This study
**pBR2-*gfp*-loop$_1$(-)-*mcherry* (TAT)**	pBR2-gfp-loop$_1$(-)-*mcherry* with the first A of TSS mutating to T	This study
**pBR2-*gfp*-loop$_1$(+)-*mcherry* (GAT)**	pBR2-gfp- loop$_1$(+)-*mcherry* with the first A of TSS mutating to G	This study
**pBR2-*gfp*-loop$_1$(-)-*mcherry* (GAT)**	pBR2-gfp-loop$_1$(-)-*mcherry* with the first A of TSS mutating to G	This study
**pBR2-*gfp*-loop$_1$(-)-*mcherry* (CCT)**	pBR2-gfp-loop$_1$(-)-*mcherry* with both A of TSS mutating to C	This study

**pBR2-*gfp*-loop$_2$(+)-*mcherry* (A1C)**	pBR2-gfp- loop$_2$(+)-*mcherry* with the first A of TSS mutating to C	This study
**pBR2-*gfp*-loop$_2$(-)-*mcherry* (CAT)**	pBR2-gfp-loop$_2$(-)-*mcherry* with the first A of TSS mutating to C	This study
**pBR2-*gfp*-loop$_2$(+)-*mcherry* (CAT)**	pBR2-gfp- loop$_2$(+)-*mcherry* with the first A of TSS mutating to T	This study
**pBR2-*gfp*-loop$_2$(-)-*mcherry* (TAT)**	pBR2-gfp-loop$_2$(-)-*mcherry* with the first A of TSS mutating to T	This study
**pBR2-*gfp*-loop$_2$(+)-*mcherry* (GAT)**	pBR2-gfp- loop$_2$(+)-*mcherry* with the first A of TSS mutating to G	This study
**pBR2-*gfp*-loop$_2$(-)-*mcherry* (GAT)**	pBR2-gfp-loop$_2$(-)-*mcherry* with the first A of TSS mutating to G	This study
**pBR2-*gfp*-loop$_2$(-)-*mcherry* (CCT)**	pBR2-gfp-loop$_2$(-)-*mcherry* with both A of TSS mutating to C	This study
**pBR2-*gfp*-loop$_1$(+)-*mcherry* (ACT)**	pBR2-gfp- loop$_1$(+)-*mcherry* with the second A of TSS mutating to C	This study
**pBR2-*gfp*-loop$_1$(-)-*mcherry* (ACT)**	pBR2-gfp-loop$_1$(-)-*mcherry* with the second A of TSS mutating to C	This study
**pBR2-*gfp*-loop$_1$(+)-*mcherry* (ATT)**	pBR2-gfp- loop$_1$(+)-*mcherry* with the second A of TSS mutating to T	This study
**pBR2-*gfp*-loop$_1$(-)-*mcherry* (ATT)**	pBR2-gfp-loop$_1$(-)-*mcherry* with the second A of TSS mutating to T	This study
**pBR2-*gfp*-loop$_1$(+)-*mcherry* (AGT)**	pBR2-gfp- loop$_1$(+)-*mcherry* with the second A of TSS mutating to G	This study
**pBR2-*gfp*-loop$_1$(-)-*mcherry* (AGT)**	pBR2-gfp-loop$_1$(-)-*mcherry* with the second A of TSS mutating to G	This study
**pBR2-*gfp*-loop$_1$(-)-*mcherry* (CCT)**	pBR2-gfp-loop$_1$(-)-*mcherry* with both A of TSS mutating to C	This study
**pBR2-*gfp*-loop$_2$(+)-*mcherry* (ACT)**	pBR2-gfp- loop$_2$(+)-*mcherry* with the second A of TSS mutating to C	This study
**pBR2-*gfp*-loop$_2$(-)-*mcherry* (ACT)**	pBR2-gfp-loop$_2$(-)-*mcherry* with the second A of TSS mutating to C	This study
**pBR2-*gfp*-loop$_2$(+)-*mcherry* (ATT)**	pBR2-gfp- loop$_2$(+)-*mcherry* with the second A of TSS mutating to T	This study
**pBR2-*gfp*-loop$_2$(-)-*mcherry* (ATT)**	pBR2-gfp-loop$_2$(-)-*mcherry* with the second A of TSS mutating to T	This study
**pBR2-*gfp*-loop$_2$(+)-*mcherry* (AGT)**	pBR2-gfp- loop$_2$(+)-*mcherry* with the second A of TSS mutating to G	This study
**pBR2-*gfp*-loop$_2$(-)-*mcherry* (AGT)**	pBR2-gfp-loop$_2$(-)-*mcherry* with the second A of TSS mutating to G	This study
**pBR2-*gfp*-loop$_2$(-)-*mcherry* (CCT)**	pBR2-gfp-loop$_2$(-)-*mcherry* with both A of TSS mutating to C	This study

*Note: pBR2 is the abbreviation of pBBR1MCS-2. pBBR1MCS-2 is a low copy-number plasmid with a *lac* promoter and a T7 terminator.

Supplementary Table 10. List of SEnd-seq and RNA-seq.

Phase	Type	Sample	SRA accession	Total reads	Uniquely mapped reads
log	SEnd-seq	WT-total	SRS4257704	5969978	611542
log	SEnd-seq	WT-total	SRS4257705	5110034	639422
log	SEnd-seq	WT-total	SRS4257706	5224284	669888
log	SEnd-seq	Δ*rne*-total	SRS3599271	3794984	1636414
log	SEnd-seq	Δ*rne*-total	SRS3599272	8590450	837364
log	SEnd-seq	Δ*pnp*-total	SRS4257708	5838702	731454
log	SEnd-seq	Δ*rnb*-total	SRS4257710	7889372	841406
log	SEnd-seq	Δ*rnr*-total	SRS4257712	6905020	807004
log	RNA-seq	WT-total	SRS4257718	5306296	4520791
log	RNA-seq	WT-total	SRS4257719	5446868	4561182
log	RNA-seq	WT-total	SRS4257720	5202190	4607000

Supplementary Table 11. Primers used in this study

Primer name	Sequence
p+m-F	TGAATGGCGAATGGAAATTGTAAGCG
p+m-R	CGCTTACAATTTCCATTCGCCATTCATCACTTGTACAGCTCGTCCAT
p+G-F	AGAGAAAGAGGAGAAATACTAGATGAGTAAAGGAGAAGAACTTTTCACTG
p+G-R	AGTATTTCTCCTCTTTCTCTAGATTCCACACAACATACGAGCC
fr1-R	TAAGCATGGCCACGCAGTCCAGAGAAATCGGCATTCAAGCC
fr1-F	GACTGCGTGGCCATGCTTATCT

Y_1-F	GGGGAATTTCCTCTCCGCCCGTGCATTCATCTAAAAGATTTAGTCTAGAGAAAGAGGAGAAATACTAGATGGTGAGC
Y_1-R	GCGGAGAGGAAATTCCCCTTCGCCCGTGTCAGTATTTATTTGTATAGTTCATACATGCC
N_1-F	GGGGAATTTCCTCTCCGCCCGTGCATTCATCTATCTAGAGAAAGAGGAGAAATACTAGATGGTGAGCAA
N_1-R	GCGGAGAGGAAATTCCCCTTCGCCCGTGTCAGTATTTATTTGTATAGTTCATACATGCC
Y_2-F	ACGAGCACGTTGTCTGCGACCCACCGCTTTTTAAAAGATTTAGTCTAGAGAAAGAGGAGAAATACTAGATGGTGAGCAA
Y_2-R	CGCAGACAACGTGCTCGTTGTTTATGCCGGATGCGTTATTTGTATAGTTCATACATGCC
N_2-F	AACGAGCACGTTGTCTGCGACCCACCGCTTTTTATCTAGAGAAAGAGGAGAAATACTAGATGGTGAGCAA
N_2-R	GCAGACAACGTGCTCGTTGTTTATGCCGGATGCGTTATTTGTATAGTTCATACATGCCA
CAT-F	TGTGTGGCATCTAGAGAAAGAGGAGAAATACTAGATGAGTAAAGGAGA
CAT-R	TCTTTCTCTAGATGCCACACAACATACGAGCCGGAA
ACT-F	TGTGTGGACTCTAGAGAAAGAGGAGAAATACTAGATGAGTAAAGGAGA
ACT-R	TCTTTCTCTAGAGTCCACACAACATACGAGCCGGAA
GAT-F	TGTGTGGGATCTAGAGAAAGAGGAGAAATACTAGATGAGTAAAGGAGA
GAT-R	TCTTTCTCTAGATCCCACACAACATACGAGCCGGAA
AGT-F	TGTGTGGAGTCTAGAGAAAGAGGAGAAATACTAGATGAGTAAAGGAGA
AGT-R	TCTTTCTCTAGACTCCACACAACATACGAGCCGGAA
TAT-F	TGTGTGGTATCTAGAGAAAGAGGAGAAATACTAGATGAGTAAAGGAGA
TAT-R	TCTTTCTCTAGATACCACACAACATACGAGCCGGAA
ATT-F	TGTGTGGATTCTAGAGAAAGAGGAGAAATACTAGATGAGTAAAGGAGA
ATT-R	TCTTTCTCTAGAATCCACACAACATACGAGCCGGAA
CCT-F	TGTGTGGCCTCTAGAGAAAGAGGAGAAATACTAGATGAGTAAAGGAGA
CCT-R	TCTTTCTCTAGACCCCACACAACATACGAGCCGGAA
bcp-UTR1-F	CATGATGAACAGGATGGAGTTAAGTAATGGTGAGCAAGGGCGAG
bcp-UTR1-R	AACTCCATCCTGTTCATCATGTTGGGAATTTCCACACA

	ACATACGAGCCGG
bcp-UTR2-F	CGTCGTCAATTATTCCCAACATGATGAACAGGATGGAG TTAAGTAATGGTGAGCAAGGGCGAGG
bcp-UTR2-R	GTTGGGAATAATTGACGACGTTAATACTGCCTTGTGCA TTGAGTTTCCACACAACATACGAGCCGG
eno-UTR1-F	CTAGTGACTTGAGGAAAACCTAATGGTGAGCAAGGGC GAG
eno-UTR1-R	GGTTTTCCTCAAGTCACTAGTTAAATTCCACACAACAT ACGAGCCG
eno-UTR2-F	GGCTCGTATGTTGTGTGGAATTAGAGCGGCAACGCGTA CCCT
eno-UTR2-R	CCTCGCCCTTGCTCACCATTAGGTTTTCCTCAAGTCACT AGTT
srlD-UTR1-F	GAATCTGTTAAGGAGTAAAAAATGGTGAGCAAGGGCG AG
srlD-UTR1-R	TTTTTACTCCTTAACAGATTCAAATTCCACACAACATA CGAGCCGG
srlD-UTR2-F	GGCTCGTATGTTGTGTGGAAATTTCCGGGCACTGTCCA TG
srlD-UTR2-R	CTCGCCCTTGCTCACCATTTTTTACTCCTTAACAGATTC AAAC
pcl1920-*pnp*-F	CATGGCGGCATACGCGATCCAAGCTGACGGCAGCAAT TC
pcl1920-*pnp*-R	CTGCAGGTCGACTCTAGAGGATCCGGTTGAATGAACGT CCTGTT
pcl1920-F	GATCCTCTAGAGTCGACCTGCAG
pcl1920-R	GATCGCGTATGCCGCCATG
rrlG-probe-F	TAATACGACTCACTATAGGCACAGATTGTCTGATGAAA AGTGAATAGC
rrlG-probe-R	TATTGAGGTCGGCGACTTTCAC
kgtP-probe-F	TAATACGACTCACTATAGGGTCGGCGGAGTACGTAGC
kgtP-probe-R	GGAGCGGCTGGAGCT
thic-probe-F	TAATACGACTCACTATAGGGATGCCCCATTTGCGGG
thic-probe-R	TGTTGGGCCCGGGC
slyX-probe-F	TAATACGACTCACTATAGGGGACCATTCGACAAAAGA GGTGAATCA
slyX-probe-R	GCCTCAATAATGTGGCGGTG
5'RACE-*gcvT*-C	TCCTCACCCTGATCCTCT
5'RACE-*gcvT*-F1	TCCAGCCGCCTGCATAC
5'RACE-*gcvT*-F2	ACTGGCGCATAAATGTCTGACG

5'RACE-*tolB*-C	ATCGTACTTGTCCAGATCG
5'RACE-*tolB*-F1	TGCTGGCGTTCTTGTTGGAAG
5'RACE-*tolB*-F2	TCACAGATACGGCGACCAGG
5'RACE-*fabG*-C	TGTAATCAATGGCAGCCTG
5'RACE-*fabG*-F2	TGCAACTAAATCCCGGCAGG
5'RACE-*fabG*-F3	ACTCATAACCACGCAGACTGC
